# Inherited metabolic disorders in adults: systematic review on patient characteristics and diagnostic yield of broad sequencing techniques (exome and genome sequencing)

**DOI:** 10.3389/fneur.2023.1206106

**Published:** 2023-07-25

**Authors:** Elise A. Ferreira, Mark J. N. Buijs, Robin Wijngaard, Joost G. Daams, Mareen R. Datema, Marc Engelen, Clara D. M. van Karnebeek, Machteld M. Oud, Frédéric M. Vaz, Mirjam M. C. Wamelink, Saskia N. van der Crabben, Mirjam Langeveld

**Affiliations:** ^1^Department of Paediatrics, Emma Children's Hospital, Amsterdam UMC, University of Amsterdam, Amsterdam, Netherlands; ^2^United for Metabolic Diseases, Amsterdam, Netherlands; ^3^Department of Human Genetics, Amsterdam University Medical Centers, University of Amsterdam, Amsterdam, Netherlands; ^4^Department of Human Genetics, Donders Institute for Brain, Cognition and Behavior, Radboud University Medical Center, Nijmegen, Netherlands; ^5^Medical Library (J.G.D.), Amsterdam UMC, University of Amsterdam, Amsterdam, Netherlands; ^6^Department of Endocrinology and Metabolism, Amsterdam UMC, Research Institute Gastroenterology, Endocrinology and Metabolism (AGEM), University of Amsterdam, Amsterdam, Netherlands; ^7^Department of Pediatric Neurology/Emma Children's Hospital, Academic Medical Center, University of Amsterdam, Amsterdam, Netherlands; ^8^Laboratory of Genetic Metabolic Diseases, Department of Clinical Chemistry, Amsterdam UMC, Gastroenterology, Endocrinology & Metabolism (AGEM), University of Amsterdam, Amsterdam University Medical Center, Amsterdam, Netherlands

**Keywords:** inherited metabolic disorders (IMD), metabolic, genomics, adults, diagnostics, exome sequencing, genome sequencing

## Abstract

**Background/Objectives:**

The timely diagnosis of inherited metabolic disorders (IMD) is essential for initiating treatment, prognostication and genetic testing of relatives. Recognition of IMD in adults is difficult, because phenotypes are different from those in children and influenced by symptoms from acquired conditions. This systematic literature review aims to answer the following questions: (1) What is the diagnostic yield of exome/genome sequencing (ES/GS) for IMD in adults with unsolved phenotypes? (2) What characteristics do adult patients diagnosed with IMD through ES/GS have?

**Methods:**

A systematic search was conducted using the following search terms (simplified): “Whole exome sequencing (WES),” “Whole genome sequencing (WGS),” “IMD,” “diagnostics” and the 1,450 known metabolic genes derived from ICIMD. Data from 695 articles, including 27,702 patients, were analyzed using two different methods. First, the diagnostic yield for IMD in patients presenting with a similar phenotype was calculated. Secondly, the characteristics of patients diagnosed with IMD through ES/GS in adulthood were established.

**Results:**

The diagnostic yield of ES and/or GS for adult patients presenting with unexplained neurological symptoms is 11% and for those presenting with dyslipidemia, diabetes, auditory and cardiovascular symptoms 10, 9, 8 and 7%, respectively. IMD patients diagnosed in adulthood (n = 1,426), most frequently portray neurological symptoms (65%), specifically extrapyramidal/cerebellar symptoms (57%), intellectual disability/dementia/psychiatric symptoms (41%), pyramidal tract symptoms/myelopathy (37%), peripheral neuropathy (18%), and epileptic seizures (16%). The second most frequently observed symptoms were ophthalmological (21%). In 47% of the IMD diagnosed patients, symptoms from multiple organ systems were reported. On average, adult patients are diagnosed 15 years after first presenting symptoms. Disease-related abnormalities in metabolites in plasma, urine or cerebral spinal fluid were identified in 40% of all patients whom underwent metabolic screening. In 52% the diagnosis led to identification of affected family members with the same IMD.

**Conclusion:**

ES and/or GS is likely to yield an IMD diagnosis in adult patients presenting with an unexplained neurological phenotype, as well as in patients with a phenotype involving multiple organ systems. If a gene panel does not yield a conclusive diagnosis, it is worthwhile to analyze all known disease genes. Further prospective research is needed to establish the best diagnostic approach (type and sequence of metabolic and genetic test) in adult patients presenting with a wide range of symptoms, suspected of having an IMD.

**Systematic review registration:**

https://www.crd.york.ac.uk/prospero/, identifier: CRD42021295156.

## Introduction

The field of inherited metabolic disorders (IMD) encompasses a growing number of (ultra) rare diseases, with currently over 1,450 disorders recognized (1). Many IMD are amenable to therapy, making their timely identification important, as early diagnosis and treatment initiation potentially prevents irreversible damage (2). Even in absence of specific treatment options, a diagnosis is desirable for patient and family as it provides closure of the diagnostic trajectory, information on prognosis, accurate genetic counseling and access to community services. In addition, supportive therapy aimed at relief of symptoms, stabilization of disease course, preventing or delaying complications, has greatly improved quality of life and in some disorders reduced morbidity and early mortality (3–5). Although most published studies focus on the identification of IMD in children, an estimated 36–50% of patients with IMD are diagnosed after the age of 16 years (6–8).

Diagnostic techniques to identify IMD have greatly changed over the past decades. The availability and accuracy of (un)targeted measurement of metabolites in plasma, urine and cerebral spinal fluid, have greatly improved (chromatography, electrophoresis and mass spectrometry (MS) platforms) (2, 9). Using these methods, a larger number of metabolites can be identified in shorter time and at lower costs (2). On the other hand, introduction of exome and genome sequencing (ES/GS) has also shown that IMDs can lack distinctive biomarkers in these matrices (10). These tests are referred to as “whole” exome or genomic sequencing both in literature and clinical settings. In reality, clinical ES/GS entails analysis of all known disease genes at a particular point in time or of a more restricted virtual gene panel (selected genes known to cause specific phenotypes). Complete analysis of all 22,000 genes is seldom performed. In this article we choose therefore to use the acronym ES for all forms of exome analysis and GS for all forms of genome analysis.

Combining metabolic screening or metabolomics with ES/GS can be helpful in validating or ruling out variants as disease-causing. Conversely specific metabolic profiles can provide clues for the underlying genetic or pathway defect, which facilitates targeted (re)analysis of specific regions and/or the discovery of a new disease gene (10).

The fact that the number of adults diagnosed with an IMD has increased significantly over the last couple of years, suggests that there are still many undiagnosed adult IMD patients (11). Diagnosing IMD in adulthood has its specific challenges. First the attenuated phenotypes of many IMD are far less well described in literature compared to the early onset phenotypes seen in children (6, 8, 12–15). Second, the symptoms related to the genetic disorder are often combined with acquired symptoms (resulting from factors such as obesity, smoking etc.) and difficult to distinguish. Moreover, much less is known about the yield of specific diagnostic tests, e.g., metabolomics or ES/GS, for unsolved adult patients compared to children. In addition, parents and/or siblings are often unavailable for trio sequencing (ES/GS), segregation analysis and/or validation of rare variants (8).

Several studies have been published on the implementation of genetic techniques in diagnosing IMD, however these studies mainly focus on pediatric patients. In this structured review we wanted to establish the current knowledge regarding the use of broad sequencing techniques for diagnosing IMDs in adults. We aimed to answer the following questions: (1) What is the diagnostic yield of ES and/or GS for IMD in adults with unsolved phenotypes (defined as objectifiable, unexplained signs and symptoms in one or more organ systems)? (2) What are the clinical and biochemical characteristics of adult patients diagnosed with an IMD through ES/GS? By answering these questions, we aspire to identify in which adult patients ES/GS are valuable tools for identifying IMD.

## Methods

### Systematic review protocol

#### Search strategy

**Figure 1:**
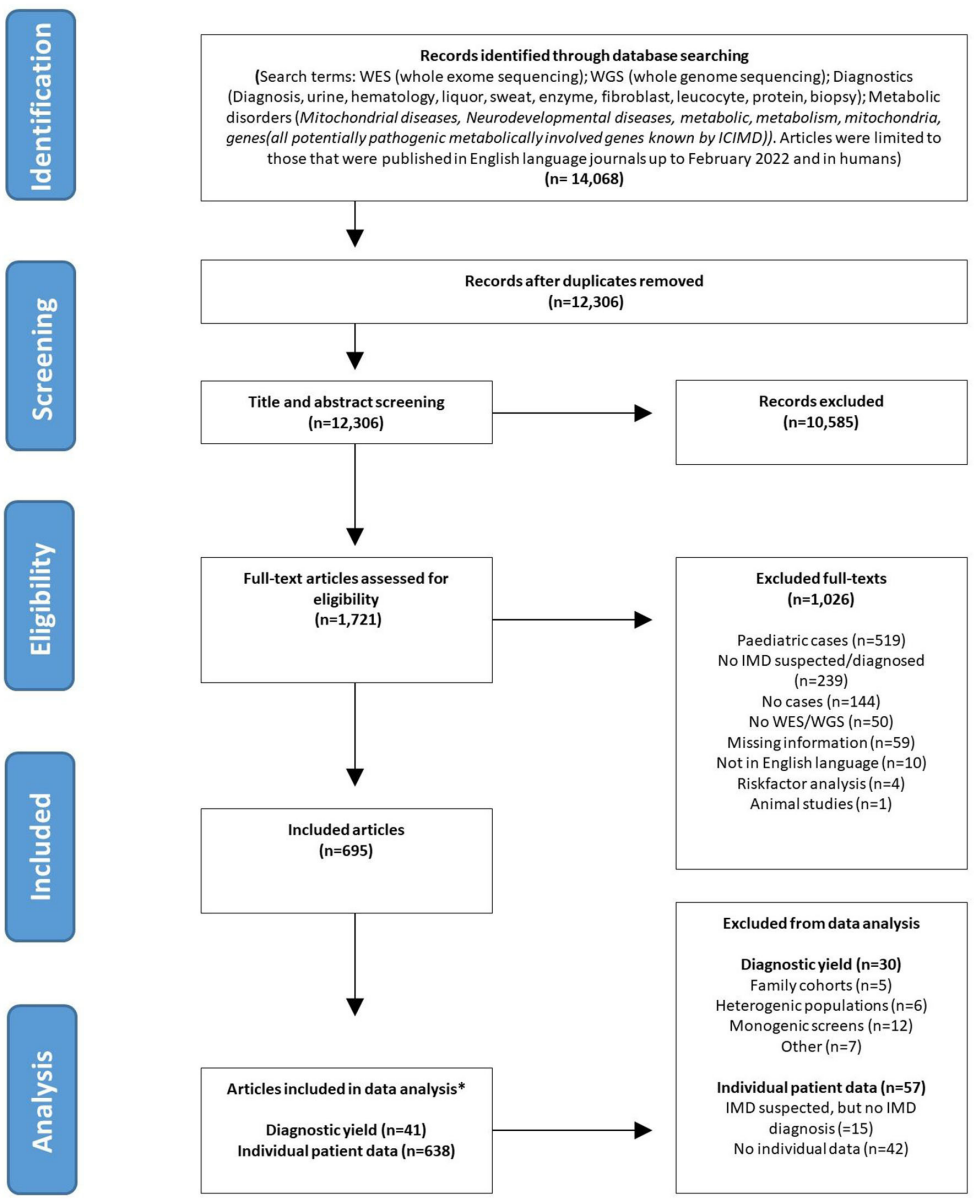
PRISMA flowchart. *Articles can be represented in both (diagnostic yield and individual patient data) analysis.

A systematic Medline and Embase search strategy was initiated by E.F., J.D. and M.L. to identify the literature of interest (the last search on 16th February 2022; Figure 1). A hand-search of bibliographies and citations of articles of interest was performed and Google Scholar was browsed for similar articles. In this manner a reference set of 50 articles was established, which was used as a template for the online search. A customized search strategy was conducted for each database with the help of a clinical librarian (J.D.). For the online search strategy, a number of keywords, which were derived from the articles in the reference set, were combined with a list of genes associated with 1,450 metabolic diseases derived from ICIMD (1) (www.icimd.org). The aim of the search was to identify all articles in which any of these disorders was diagnosed in an adult patient and either ES or GS had been part of the diagnostic tools used. The conceptual display of the multi stranded search approach for each database is as follows:

*([WES/WGS] AND [McMaster diagnosis high-sensitivity filter] AND [metabolic diseases derived from ICIMD])*

*OR*

*([WES/WGS] AND [McMaster diagnosis high-sensitivity filter] AND [unexplained phenotypes/metabolic errors])*

*OR*

*([WES/WGS] AND [metabolic diseases derived from ICIMD] AND [unexplained phenotypes/metabolic errors])*

*NOT*

*([animals] OR [irrelevant terms derived from VOS clusters])*

See Supplementary Data Sheet 1 for comprehensive and detailed search strategies (Supplementary Data Sheet 1: Comprehensive and detailed search strategies).

#### Study population and selection of studies

Papers identified by the search strategy were uploaded in Rayyan for screening (16). Full text versions of all records deemed eligible on the basis of title and abstract, were searched with two electronic databases or alternatively searched via Google Scholar. The retrieved full texts were reviewed for inclusion. One co-author (E.F.) independently reviewed the 12,306 publications that emerged from the searches for potential inclusion in the review. Because of the magnitude of the search outcome, a second author (M.B.) randomly reviewed a subset of these articles (*n* = 1,600) in order to validate reviewer E.F.'s outcomes. Discrepancies between the reviewers were resolved by consensus. All article types were included, apart from those published as an abstract only. Only articles studying adult patients were selected. Adults were defined as: patients of ≥16 years of age at time of diagnosis or at time of ES and/or GS. The age of onset of symptoms could be before 16 years of age. The following articles were excluded: those reporting on *in vitro* studies or animal studies, duplicates and those dealing with pediatric populations only or in which the data of adults could not be separated from those of children. Articles that did not include patients that were either diagnosed with, or suspected of having an IMD, were excluded. Publications in English were included (Figure 1). The protocol for this literature search was registered in the Prospero database, registration number [CRD42021295156] (https://www.crd.york.ac.uk/prospero/).

### Data extraction

A standardized form was designed in CASTOR EDC to collect information systematically. Data were extracted by 3 reviewers (E.F., M.B. and R.W.) independently (17).

*Data were extracted in two ways:*

1. **Diagnostic yield**: For this analysis we included all studies reporting on more than 10 individuals presenting with similar unexplained symptoms, classified using HPO terms (e.g., abnormalities of the neurological system or abnormalities of the eye). We excluded studies reporting on a single family, reporting on a heterogenic patient population and studies that did not report on negative ES/GS results in patients.
2. **Single cases:** to determine the phenotype of adults diagnosed with an IMD, we extracted information on an individual case level from all included study types (case reports, case series, cohort studies). For each participant the following information was collected: age of onset symptoms, age at diagnosis; gender; ethnicity; phenotype [using HPO symptom categories to categorize the symptoms, (18)]; whether metabolite testing was performed and if they were abnormal; involved IMD gene; mode of inheritance; genetic variant classification reported by the authors (benign, variant of unknown significance, likely pathogenic, pathogenic); DNA analysis technique used (ES and/or GS); techniques used to validate pathogenicity of the variant (e.g., metabolite measurements, functional assays); affected family members. By metabolite measurements we refer to the measurement of a subset or all metabolites, i.e., acyl carnitines, amino acids, organic acids in plasma, urine and/or CSF, often referred to as “metabolic screening” and measurement of a specific metabolite or set of metabolites relevant to validate the pathogenicity of a genetic variant. We categorized the observed metabolic abnormalities into groups according to the ICIMD (e.g., abnormalities in fatty acid and ketone body metabolism). Since the largest number of studies concerned patients with an unexplained neurological phenotype, we decided to further specify this group into the following categories: extrapyramidal/cerebellar symptoms, peripheral neuropathy, pyramidal tract symptoms/myelopathy, epilepsy and intellectual disability/dementia/psychiatric symptoms. The HPO category “metabolic/homeostasis abnormalities” was further split into dyslipidemia, iron accumulation, abnormalities in routine clinical biochemistry and abnormalities in full blood count. A number of articles contained patient data that could be used both in the diagnostic yield analysis and in the single cases analysis (data could be extracted at a single patient level).

#### Classification IMD genes and metabolites

Genes identified as causative in the published articles were categorized as IMD-causing if they were present in the ICIMD database (1). Both the genetic variants as well as the metabolite abnormalities were categorized in 24 metabolic pathway subgroups based on the ICIMD classification system (e.g., abnormalities of complex molecule degradation or neurotransmitter disorders).

#### Statistical analysis

The diagnostic yield was calculated as the proportion of adults diagnosed with an IMD of the total cohort in which ES/GS was performed. Frequencies of symptoms, demographics, genes and metabolite abnormalities, were calculated as the percentage of the total 1,426 single cases. The average diagnostic delay was calculated by extracting the number of years at age diagnosis from the age at onset of first symptoms. We performed all analyses using IBM SPSS statistics, version 26 and R version 4.2.1 (2022-06-23 ucrt).

## Results

### Search results

As shown in Figure 1, a total of 14,068 articles were identified in the initial search. After removal of duplicates, 12,306 articles remained. An additional 10,585 were excluded after title and abstract screening because they assessed the wrong study population, the abstract was not available, the article was not written in English or for other reasons. There were no discrepancies between the results of the two authors that screened the abstracts. After full-text assessment of the remaining 1,721 articles, 695 articles fulfilled the inclusion criteria and were included in this systematic review (reasons for exclusion of the other 1,026 articles listed in Figure 1).

The 695 reviewed articles, reported on a total number of 27,702 adult patients in whom ES/GS was performed. A total of 2,210 patients were diagnosed with an IMD through ES and/or GS. The majority of these patients (1,647 cases; 75%), were not suspected of having an IMD prior to sequencing. Data could be extracted at an individual level for 1,426 adult IMD patients.

### Diagnostic yield in patient cohorts

A total of 41 articles reported on patient cohorts (>n = 10), with a total of 5,574 ES/GS sequenced patients of whom 590 adults were diagnosed with IMD. There were large differences in the total number of patients included per cohort for the different symptom categories, ranging from 4,100 patients with neurological symptoms to 103 patients with ophthalmological symptoms. The diagnostic yield for diagnosing IMD in adults with ES and/or GS in the largest included cohorts was: 11% (486/4,100) for patients with abnormalities of the nervous system; 10% (32/310) for dyslipidemia; 9% (5/57) for diabetes and 7% (52/762) for patients presenting with a cardiovascular phenotype (e.g., early myocardial infarction or unexplained pulmonary embolism). For patients with ophthalmological symptoms, the diagnostic yield of ES/GS was very low (1%; 1/103), but is important to note that this cohort consisted of patients with high myopia only (19).

**Figure 2:**
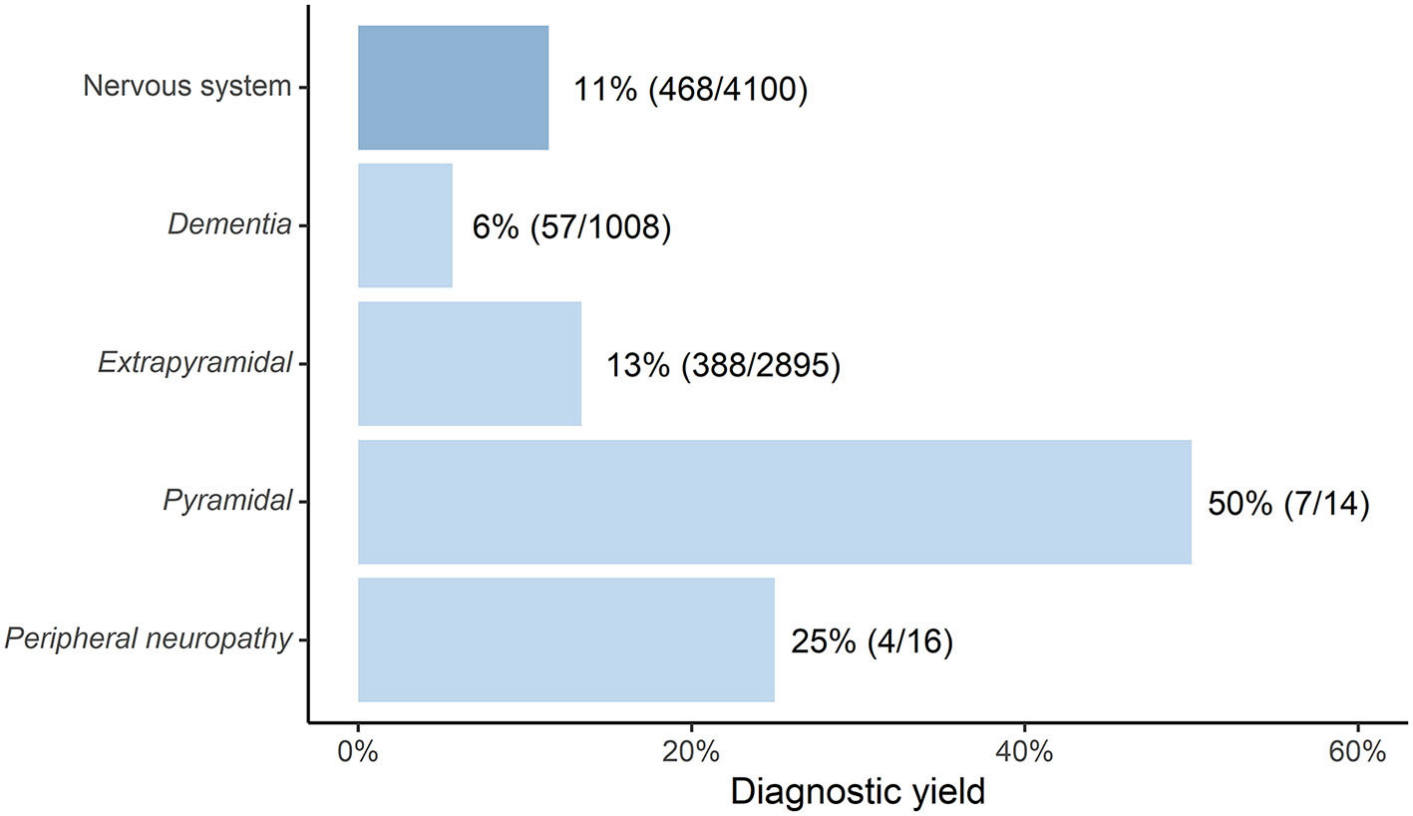
Diagnostic yield of ES and/or GS in diagnosing IMD in adult cohorts (*n* = > 10) with neurological symptoms [depicted as “Percentage(adult diagnosed with IMD/total adults sequenced with ES/GS)”].

Within the cohort presenting with abnormalities of the nervous system, the largest patient groups were those with extrapyramidal signs (*n* = 2,895) and with dementia (*n* = 1,008), with an IMD diagnostic yield of 13% and 6% respectively after ES/GS. The IMD diagnostic yields for pyramidal signs (50%) and peripheral neuropathy (25%) are much less reliable since these cohorts were very small (14 and 16 patients, respectively). For a full overview of the diagnostic yield of ES/GS for the nervous system, (see Figure 2), for all included studies and the diagnostics yield of ES/GS for other organ systems see Supplementary Figure 1.

### Single case analysis of adults diagnosed with and IMD through ES and/or GS

**Figure 3:**
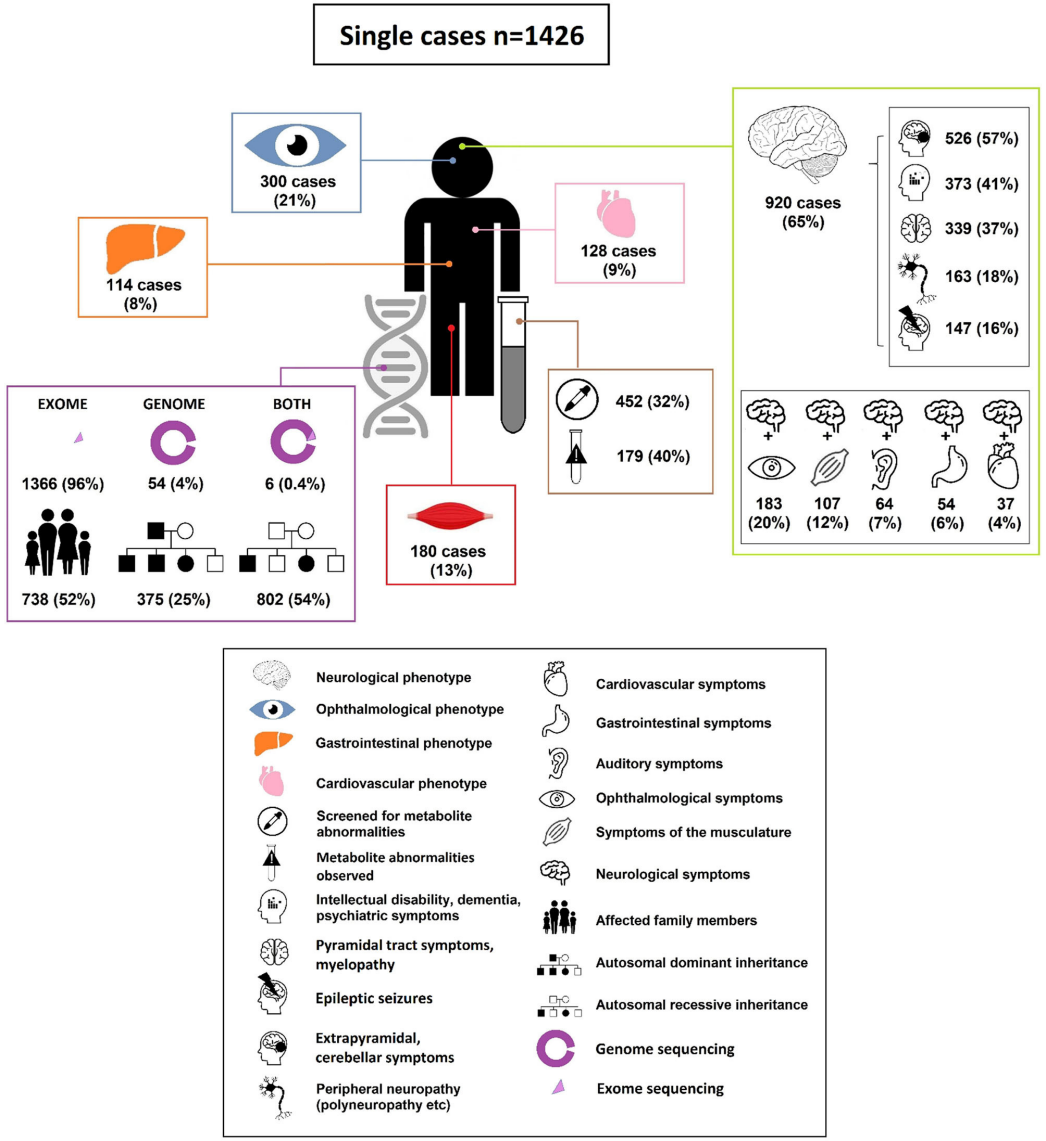
Characteristics of adults diagnosed with an IMD through ES and GS.

Data of 1,426 patients could be extracted from 638 articles on an individual case level (general characteristics outlined in Table 1 and Figure 3). Of these, 634 patients (44%) were female, 697 patients (49%) were male and for 95 patients (7%) we could not establish the gender from the records. The largest proportion of patients were of Asian origin (414 cases; 29%), followed by those of European (351 cases; 25%) and Middle Eastern/Arab (161 cases; 11%) descent. The median age at the onset of symptoms was 20 years (range 0–80 years) and median age at genetic diagnosis 35 years (range 16–90 years). From these numbers an average diagnostic delay of 15 years per adult IMD patient can be deduced.

**Table 1:** Adult IMD patient characteristics.

	**Patient characteristic (*n* = 1,426)**	**Frequency (number)**	**Percentage (%)**
**Gender**			
	Female	634	45%
	Male	697	49%
	Not reported	95	7%
**Ethnicity**			
	Asian	414	29%
	European	351	25%
	Middle Eastern/Arab	161	11%
	North-American	70	5%
	South American (incl Hispanic and Latino)	27	2%
	African and African American	25	2%
	Not reported	378	27%
Age (years)	Median onset of symptoms^*^	20 (range 0-−80 years)	
	Median age of diagnosis	35 (range 16-−90 years)	
Average diagnostic delay^Δ^		15 years	
**Multiple system involvement (2 or more organ**			
**systems involved; % of all cases)**			
	Yes	674	47%
	No	752	53%

^*^30% missing data. ^Δ^The number of years between age at onset first symptom presentation until diagnosis.

In the 1,426 cases the following genetic diagnostic technique was used: ES (1,366 cases; 96%), GS in (54 cases; 4%), both ES and GS (6 cases; 0.4%). In 924 cases (65%) the IMD diagnosis was based on ES or GS alone. In 66 cases (5%), the IMD was confirmed by a matching metabolite pattern after identifying a potential causal pathogenic variant. In 224 cases (16%) ES/GS results were validated using a functional assay. In the remainder of cases the diagnosis was confirmed by various imaging modalities (10 cases; 0.7%), electromyography (31 cases; 2%) or extensive eye examination (OCT, fundoscopy etc.) (79 cases; 6%). In the remaining 6% of cases, more than two validation tools were applied. Mode of inheritance of the diagnosed disorders was predominantly autosomal recessive (802 cases; 54%), followed by autosomal dominant (75 cases; 25%); x-linked inheritance (40 cases; 3%) or *de novo* occurrence (29 cases; 2%). In more than half of the cases (738 cases; 52%), cascade screening led to diagnosis of the same IMD in a relative.

The top 5 most common symptoms in the 1,426 adults diagnosed with an IMD through ES/GS were: neurological symptoms 65% v= 920); ophthalmological symptoms, 21% (*n* = 300); muscle symptoms, 13% (*n* = 180); cardiovascular symptoms, 9% (*n* = 128) and hepato-gastroenterological symptoms 8% (*n* = 114). In 110 (8%) of the cases, patients showed “metabolic/homeostasis abnormalities” that were observed in routine clinical investigations (e.g., abnormalities routine clinical biochemistry, dyslipidemia). In 674 cases (47%) multiple organ system involvement (two or more systems) was observed.

#### Diagnoses

ES/GS identified variants in a total of 446 unique IMD genes. The majority of IMD gene variants were classified by authors as likely pathogenic or pathogenic (Class 4–5) according to ACMG guidelines (1,336 cases; 90%) (20). These (likely) pathogenic variants were most often identified in IMD genes associated with: different aspects of mitochondrial form and function: 19% (287 cases); lipid metabolism: 13% (196 cases); complex molecule degradation 13% (190 cases); neurotransmitter metabolism 9% (136 cases) and function of organelle biogenesis, dynamics and interactions: 7% (109 cases).

The characteristics of patients presenting with abnormalities in the most commonly observed symptom groups: neurological symptoms, ophthalmological symptoms, muscle symptoms, cardiovascular symptoms and hepato/gastro-intestinal symptoms, are discussed below.

#### Neurological symptoms

**Figure 4:**
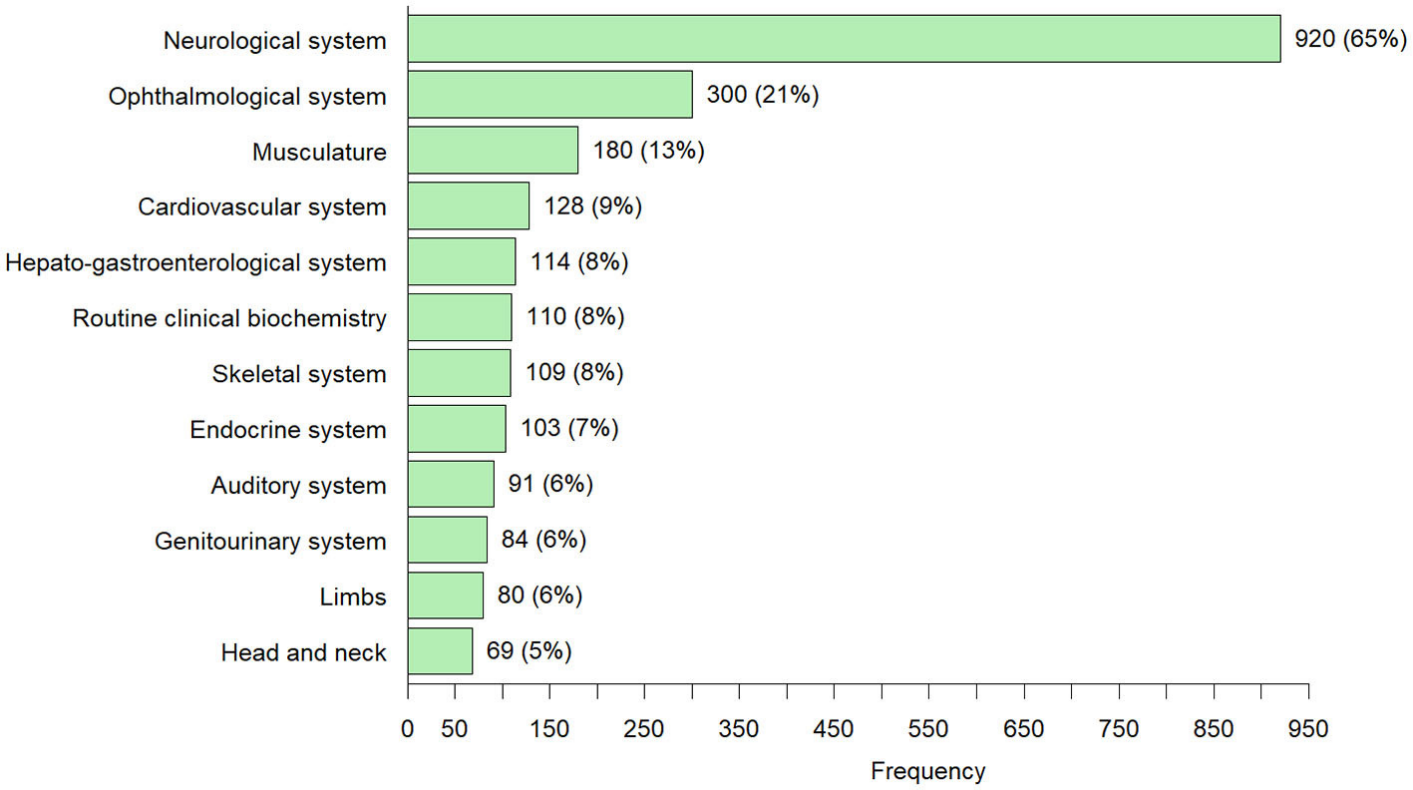
Overview of all symptoms that occurred in ≥5% of the 1,426 adults diagnosed with an IMD through ES and/or GS [number of cases (percentage)]—Individuals can have more than one symptom from the different HPO symptom groups.

The majority of the patients diagnosed with an IMD through ES/GS in adulthood presented with neurological symptoms (920/1,426 cases; 65%) (Figure 4). In roughly half of the patients with a neurological phenotype (471/920 cases; 51%) there was involvement of one or more other organ systems (ophthalmological symptoms 20%, muscle symptoms 12%, auditory symptoms 7%, hepato-gastroenterological symptoms 6% and cardiovascular symptoms 4%, for full overview see Figure 3; Supplementary Table 1). In 20% of patients with neurological symptoms, dysmorphic features were observed (ranging from pes cavus/equinovarus to specific dysmorphic facial features). The median onset of neurological symptoms was 20 years (range 0–80 years) and the median age of diagnosis was 35 years (range 16–90 years).

**Figure 5:**
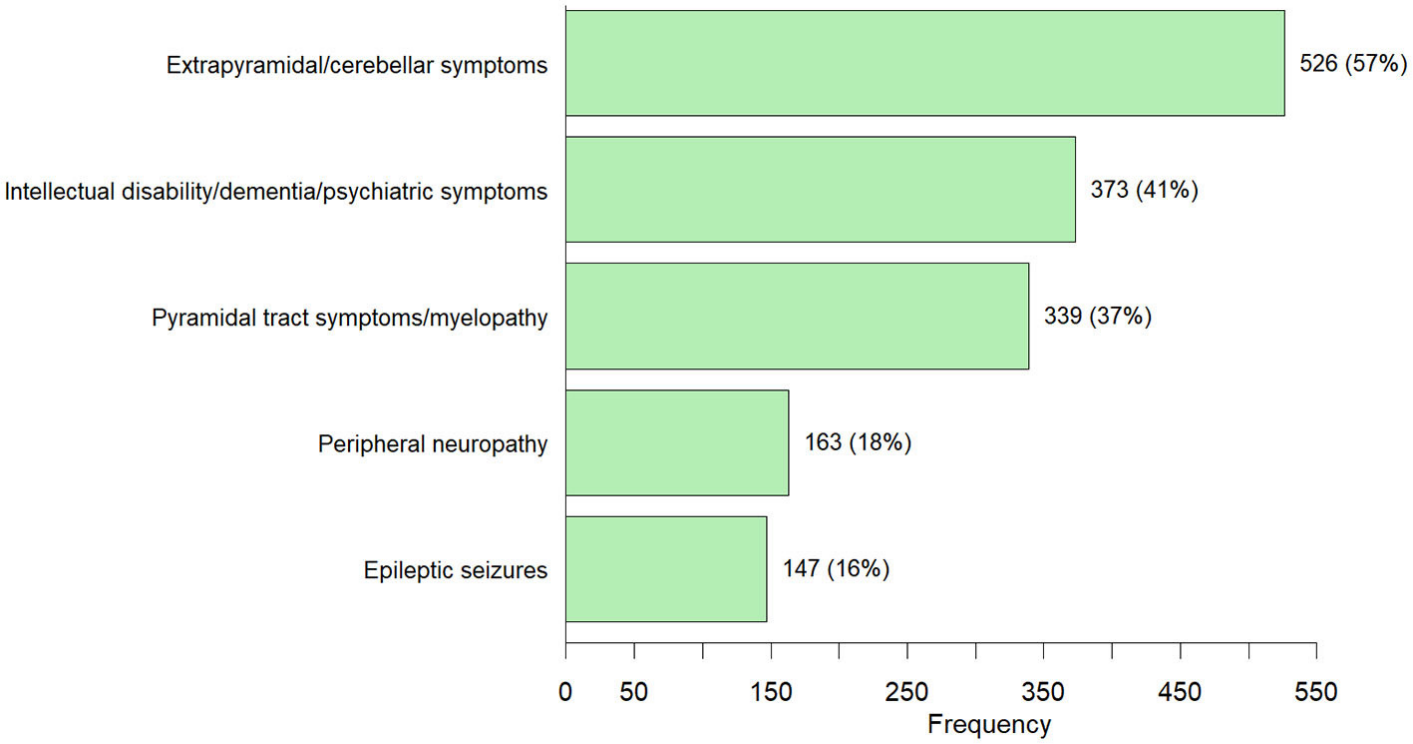
Neurological symptoms observed in 920 adults diagnosed with an IMD through ES/GS [number of cases (percentage)]**—**Individuals can have more than one neurological symptom from the different neurological subgroups.

The top 5 IMD genes reported in the patients presenting with neurological symptoms were *SPG7*: 5% (50 cases), *LRRK2*: 5% (41 cases), *SACS* 4% (40 cases), *KIF1A* 3% (26 cases) and *SPG11* 3% (23 cases). According to the ICIMD classification, *SPG7* and *SACS* are both involved in mitochondrial function. Affected *LRRK2* and *KIF1A* genes cause disorders of neurotransmission. *SPG11* is a causative gene for disorders of complex molecule degradation. Metabolite measurements were performed in 27% (248/920) of neurological patients. In 21% (51/248) of the neurological patients who underwent metabolite testing, the abnormalities were in the same metabolic pathway as the identified causative genetic variant. The observed neurological symptoms could be classified in the following subgroups: extrapyramidal/cerebellar symptoms: 57% (526 cases); intellectual disability/dementia/psychiatric symptoms 40% (372 cases); pyramidal symptoms 37% (339 cases); peripheral neuropathy 18% (163 cases) and epilepsy 16% (147 cases) (Figure 5). Multiple types of neurological signs/symptoms could be present in a single patient. Detailed information on the frequency of the genetic variants in patients with neurological symptoms can be found in Supplementary Table 2.

#### Ophthalmological symptoms

The 300 (21%) of the 1,426 patients diagnosed with an IMD through ES/GS in adulthood, exhibited ophthalmological symptoms (retinitis pigmentosa, optic atrophy, retinal dystrophy etc.). A minority of these patients solely portrayed ophthalmological symptoms (22%). In 61% of these patients, additional neurological symptoms were observed. Variants were detected in 128 different IMD genes, all of the individual genes presented with a low frequency ( ≤ 5%). Metabolite measurements were performed in a minority of patients (27%, 80/300). In 25% (20/80) of patients, the metabolite abnormalities were found in the same metabolic pathway as the causative genetic variant. Information on the frequency of genetic variants and metabolite abnormalities observed in patients with ophthalmological symptoms can be found in Supplementary Table 2.

#### Muscle symptoms

The 180 (13%) of the 1,426 patients diagnosed with an IMD through ES/GS in adulthood displayed muscle symptoms. In 19% of these patients this was the only affected organ system. In 60% of patients with muscle symptoms, accessory neurological symptoms were observed. Other frequently accompanying symptoms came from the ophthalmic system (30 cases, 17%), cardiovascular system (20 cases, 11%), skeletal system (19 cases, 11%) and auditory system (11 cases, 6%). Variants were detected in 493 different IMD genes, all of the individual genes presented with a low frequency ( ≤ 5%). Metabolite measurements were performed in 51% (91/180) patients. In 22% (20/91) of patients, the metabolite abnormalities were found in the same metabolic pathway as the causative genetic variant. Information on the frequency of the genetic variants and metabolite abnormalities observed in patients with muscle symptoms can be found in Supplementary Table 2.

#### Cardiovascular symptoms

In 128 (9%) of all patients diagnosed with an IMD through ES/GS in adulthood, abnormalities of the cardiovascular system were observed (i.e., cardiomyopathy, hypertension, early atherosclerosis). In the majority of cases the symptoms were part of a multisystem disease (72%). In 128 cases (29%) additional neurological symptoms were observed. Metabolite measurements were performed in 56% (71/128) of the patients. In 49% (35/71) of patients, the metabolite abnormalities were found in the same metabolic pathway as the identified causative genetic variant. Information on the frequency of the genetic variants and metabolite abnormalities observed in patients with cardiovascular symptoms can be found in Supplementary Table 2.

#### Hepato-gastroenterological symptoms

The 114 out of the 1,426 IMD patients (8%) suffered from hepato-gastroenterological symptoms (predominantly abnormalities of the liver, liver function or the pancreas). Multisystem involvement was observed in 81% of these patients and 47% of patients had additional neurological symptoms. Metabolite measurements were performed in 46% (53/114) of the patients. In 62% (33/53) of patients, the metabolite abnormalities were found in the same metabolic pathway as the causative genetic variant. Information on the frequency of the genetic variants and metabolite abnormalities in patients with hepato-gastroenterological symptoms can be found in Supplementary Table 2.

## Discussion

We present the first comprehensive systematic literature review on the use of ES and/or GS in diagnosing IMD in adults with unexplained phenotypes. The average time between onset of symptoms and time of diagnosis in patients diagnosed with an IMD through ES/GS in adulthood included in this review, was 15 years. In the majority of patients diagnosed with IMDs, an IMD was not suspected prior to sequencing. This emphasizes the need for more awareness and improved recognition of these disorders in clinic. Using the findings from this review we are able to provide some guidance to improve diagnostic strategies. First, unexplained neurological symptoms should prompt investigations toward the presence of an IMD. Especially movement disorders, spasticity and cognitive symptoms are found in a large proportion of patients with an IMD. About half of the patients showed isolated neurological signs and symptoms, while the other half presented with a multisystem disease. Especially combinations of ophthalmological and neurological symptoms were frequently observed in patients with an IMD, and therefore neurologists and ophthalmologists should include metabolic disorders to their differential diagnosis when encountering such patients in clinic. Our calculations of the diagnostic yield of ES/GS in diagnosing IMD, confirm the notion that it is worthwhile to look for IMDs in adult patients with unexplained extrapyramidal symptoms (diagnostic yield 13%), which is higher than previous estimates of the diagnostic yield of ES/GS for diagnosing IMD in general (9,8%) (21). In contrast, the diagnostic yield for IMD with early onset dementia (not in the context of intellectual disability) was relatively low (6%). For the other neurological subgroups, the diagnostic yield could not be reliably calculated due to the small sample size in the relevant studies (range 14–16 patients).

Second, for the majority of IMD patients (72–88%) presenting with ophthalmological, cardiovascular, muscle or hepato-gastroenterological signs and symptoms, the symptoms were part of a multisystem disorder. Thus, specialists (ophthalmologists, cardiologists, gastroenterologists) need to be on the lookout for signs and symptoms suggestive for impairments outside of the field of their expertise and refer patients for additional testing to uncover the complete phenotype. Detailed phenotyping is crucial for the effective interpretation of ES/GS data and will contribute to exclusion of non-pathogenic variants that do not match the phenotype.

The four most commonly detected affected genes in the patients presenting with a neurological phenotype were SPG7, LRRK2, SACS and KIF1A. As pathogenic variants in these genes are not immediately associated with metabolic disorders, this warrants some discussion regarding the definition of an inherited metabolic disorder. One way of thinking about this, is categorizing IMDs in three groups: (1) Classical IMD: a disorder with evident biochemical abnormalities in easy to reach matrices (blood, urine) (e.g., elevated plasma ammonia and urine orotic acid in patients with OTC deficiency). These were underrepresented in the current review since they will be relatively easily diagnosed and thus not fall under the definition of unexplained phenotype. (2) IMD caused by impairments of metabolic pathways intrinsic to the pathophysiology of a disease, without abnormalities in currently available biochemical laboratory tests in easy to reach matrices (e.g., polyglucosan body myopathy type 2 (caused by pathogenic variants in *GYG1*), an IMD without detectable biomarkers in blood or urine, but at tissue level is clearly caused by polyglucosan accumulation due to impaired branching of glycogen) (22). (3) Disorders caused by pathogenic variants in genes that affect metabolic processes, but which function is not fully known or they are indirectly involved in metabolic pathways. This could also apply for the above mentioned genes: SPG7 for example; the exact function of the affected paraplegin protein in SPG7 is unknown, but defective paraplegin is associated with accumulation of mitochondrial DNA damage and multiple respiratory chain deficiencies) (23). SACS is associated with lysosomal functioning (24). LRRK2 and KIF1A are associated with (amongst others) lipid metabolism. For more details on the effect of pathogenic variants in these genes see Supplementary Table 2 (Tab: *frequently affected genes*).

There are challenges in interpreting ES/GS data, some of which are specific to the adult patient population. Coverage of some areas in ES are still low and in cases with high clinical suspicion of a specific disease, additional genetic testing might be necessary. Due to high age, for many adult patients, parents and relatives are often not available for genetic analysis. Thus segregation analysis to proof pathogenicity is often impossible or limited. To limit incidental findings or detection of variants of unknown significance (VUS), analyzing gene panels is often preferred since here only a certain set of genes is assessed. The downside of this approach is that (likely) pathogenic variants outside the gene panel are not reported. The panels are generally organ system/symptom specific, making them less applicable in case of multisystem involvement. Additionally, panels need to be curated/updated, thus are dependent on the publication of cases with new phenotypes or the discovery of new disorders and gene panels differ between hospitals and commercial laboratories. Furthermore, adults potentially suffer from genetic disorders for which the adult-onset type has only recently been recognized and described. The 100,000 genomes pilot study on rare-disease diagnosis from the UK, in which the majority (74%) of included patients were adults, showed that 40% of the diagnoses were established after variant analysis outside of the first applied gene panels (25). This is in line with the findings in our review in which a wide range of genes were discovered for each of the symptom groups, which could not all be included in a single gene panel. Therefore, if a limited virtual gene panel does not yield a diagnosis in those adult patients with a high suspicion of a genetic (metabolic) disorder, the genetic diagnostic approach should be expanded to sequencing all known disease genes. Because of the complexity of interpreting rare genetic variants and the possibility of incidental findings, we recommend to apply this approach in a multidisciplinary setting, involving a (pediatric) IMD specialist, a neurologist, clinical geneticist and (molecular and metabolic) laboratory specialist. For further diagnostic suggestions in case of a “negative exome,” we refer to the work of Wortmann et al. (26).

An important question in establishing the most efficient diagnostic trajectory in adult patients suspected of having an IMD, is the timing of metabolite measurements (metabolic screening). In the current study metabolite screening was only applied in a minority of patients (32%) diagnosed with an IMD (27%-51%, for the different symptom groups), therefore the exact diagnostic yield of metabolic screening for diagnosing IMD, cannot be given. In addition, it was not always clear if these measurements were performed before or after ES/GS. Our results do suggest that measuring metabolites may be specifically useful in patients presenting with cardiovascular or hepato-gastro-intestinal symptoms, since relatively high percentages of relevant metabolite abnormalities (49% and 62%, respectively) were found in these groups.

### Limitations of the current review

There are several risks of bias in our dataset. An important limitation is the retrospective design of this study. Especially recall bias and observer bias may have occurred, for example not reporting the full range of symptoms or not describing all detected variants in the publications reporting on the included cases. Additionally, the fact that we encountered a large number of adult IMD patients presenting with neurological symptoms could be because ES and/or GS is more often applied in adults presenting with this type of symptoms. Also, neurological symptoms could be more noticeable by both patients and physicians. Patients with less pronounced symptoms (e.g., night blindness), are not as likely to receive DNA sequencing until additional symptoms arise, symptoms deteriorate or more relatives appear affected. The scope of our search was to find adult IMD cases diagnosed through ES and/or GS. These DNA techniques are often a last resort, reserved for cases with an unexplained (metabolic) phenotype. Cases with clear leads from metabolite screens, where the diagnosis was confirmed by Sanger sequencing or targeted gene panels, do not appear in this data collection. The current data analysis gives us a perspective on diagnosing IMD in adults based on data of the past decade (since broad sequencing techniques were applied in clinic). However, both the technical aspects as well as the interpretation of data is rapidly evolving (changing panels, new pipelines with better accuracy, novel genes, different ways of interpretation and validating VUS etc.) which means that a study performed in 2030 will yield different results than our current study.

In summary, exome and/or genome sequencing is likely to yield an IMD diagnosis in adult patients presenting with an unexplained neurological phenotype, as well as in patients with a phenotype involving multiple organ systems. If a gene panel does not yield a conclusive diagnosis, it is worthwhile to analyze all known disease genes albeit in a multidisciplinary setting in a center of expertise in rare (inherited metabolic) diseases. Further prospective research is needed to establish the best diagnostic approach (type and sequence of metabolic and genetic test) in adult patients presenting with a wide range of symptoms, suspected of having an IMD.

## Data availability statement

The original contributions presented in the study are included in the article/Supplementary material, further inquiries can be directed to the corresponding author.

## Author contributions

EF: designing study, writing systematic review protocol, defining search term, screening of abstracts and article, designing database, data extraction, data analysis, data interpretation, and drafting manuscript. MB: screening of abstracts and articles, data extraction, data analysis, and reviewing manuscript. RW: data extraction, data analysis, and reviewing manuscript. JD: defining search term and conducting search. MD: designing database and data analysis. ME, FV, and MW: designing study and reviewing manuscript. CK and MO: reviewing manuscript. SC: designing of study, data interpretation, and reviewing manuscript. ML: conceptualizing and designing study, defining search term, designing database, data interpretation, and drafting manuscript. All authors contributed to the article and approved the submitted version.
