# Supplementary material for: Inherited metabolic disorders in adults: systematic review on patient characteristics and diagnostic yield of broad sequencing techniques (exome and genome sequencing)
Source: Front Neurol. 2023 Jul 25;14:1206106. doi: 10.3389/fneur.2023.1206106 (PMC10408679; doi:10.3389/fneur.2023.1206106)
Supplement: Supplementary file 3 [file Data_Sheet_1.PDF]

Appendix 1| Search

Ovid MEDLINE(R) ALL <1946 to February 15, 2022>
Search date: 16 February 2022

#	Searches	Results
1	exp whole genome sequencing/	14596
2	(wgs or (whole genom* adj5 sequenc*)).ab,kf,ti.	31222
3	(wes or ((exome or Transcriptome) adj3 sequenc*)).ab,kf,ti.	31202
4	or/1-3 [WES/WGS]	64793
5	(sensitiv: or diagnos:).mp. or di.fs. [McMaster diagnosis high sensitivity filter]	6856916
6	("A4GALT" or "AAGAB" or "AARS1" or "AARS2" or "AASS" or "ABAT" or "ABCA1" or "ABCA12" or "ABCB11" or "ABCB4" or "ABCB6" or "ABCB7" or "ABCC2" or "ABCC6" or "ABCC8" or "ABCC8" or "ABCD1" or "ABCD3" or "ABCD4" or "ABCG5" or "ABCG8" or "ABHD12" or "ABHD5" or "ACACA" or "ACACB" or "ACAD8" or "ACAD9" or "ACADM" or "ACADS" or "ACADSB" or "ACADVL" or "ACAT1" or "ACAT2" or "ACBD5" or "ACER3" or "ACO2" or "ACOX1" or "ACOX2" or "ACSF3" or "ACSL4" or "ACY1" or "ADA" or "ADA2" or "ADAR" or "ADARB1" or "ADAT3" or "ADCK2" or "ADK" or "ADSL" or "ADSS1" or "AFG3L2" or "AGA" or "AGK" or "AGL" or "AGMO" or "AGPAT2" or "AGPS" or "AGXT" or "AGXT2" or "AHCY" or "AICDA" or "AIFM1" or "AIMP1" or "AIMP2" or "AK1" or "AK2" or "AK7" or "AKR1C2" or "AKR1D1" or "AKT2" or "ALAD" or "ALAS2" or "ALAS2" or "ALB" or "ALDH18A1" or "ALDH1L2" or "ALDH3A2" or "ALDH4A1" or "ALDH5A1" or "ALDH6A1" or "ALDH7A1" or "ALDOA" or "ALDOB" or "ALG1" or "ALG11" or "ALG12" or "ALG13" or "ALG14" or "ALG2" or "ALG3" or "ALG6" or "ALG8" or "ALG9" or "ALOX12B" or "ALOXE3" or "ALPL" or "AMACR" or "AMN" or "AMPD1" or "AMPD2" or "AMPD3" or "AMT" or "ANGPTL3" or "ANPEP" or "AP1B1" or "AP1S1" or "AP1S2" or "AP2S1" or "AP3B1" or "AP3B2" or "AP3D1" or "AP4B1" or "AP4E1" or "AP4M1" or "AP4S1" or "AP5Z1" or "APOA1" or "APOA5" or "APOB" or "APOC2" or "APOC3" or "APOE" or "APOE" or "APOO" or "APOPT1" or "APPL1" or "APRT" or "AR" or "ARCN1" or "ARFGEF2" or "ARG1" or "ARSA" or "ARSB" or "ARSG" or "ASAH1" or "ASL" or "ASNS" or "ASPA" or "ASS1" or "ATAD1" or "ATAD3A" or "ATG5" or "ATIC" or "ATP13A2" or "ATP5F1A" or "ATP5F1D" or "ATP5F1E" or "ATP5MD" or "ATP5PO" or "ATP6AP1" or "ATP6AP2" or "ATP6V0A2" or "ATP6V1A" or "ATP6V1E1" or "ATP7A" or "ATP7B" or "ATP8A2" or "ATP8B1" or "ATPAF2" or "AUH" or "B3GALNT2" or "B3GALT6" or "B3GAT3" or "B3GLCT" or "B4GALNT1" or "B4GALT1" or "B4GALT7" or "B4GAT1" or "BAAT" or "BBOX1" or "BCAP31" or "BCAT2" or "BCKDHA" or "BCKDHB" or "BCKDK" or "BCS1L" or "BLK" or "BLOC1S3" or "BLOC1S6" or "BLVRA" or "BMP6" or "BMS1" or "BOLA3" or "BPNT2" or "BSCL2" or "BSCL2" or "BTD" or "C12orf65" or "C1GALT1C1" or "C1QBP" or "CA5A" or "CAD" or "CANT1" or "CARS1" or "CARS2" or "CAT" or "CBLIF" or "CBS" or "CCDC115" or "CCS" or "CD320" or "CEP89" or "CERS1" or "CERS2" or "CERS3" or "CETP" or "CHAT" or "CHCHD10" or "CHCHD2" or "CHKB" or "CHRNE" or "CHST11" or "CHST14" or "CHST3" or "CHST6" or "CHSY1" or "CLCN2" or "CLN3" or "CLN5" or "CLN6" or "CLN8" or "CLP1" or "CLPB" or "CLPP" or "CLPX" or "CLTC" or "CMPK2" or "CNDP1" or "COA3" or "COA5" or "COA6" or "COA7" or "COASY" or "COG1" or "COG2" or "COG4" or "COG5" or "COG6" or "COG7" or "COG8" or "COL4A3BP" or "COPA" or "COPB2" or "COQ2" or "COQ4" or "COQ5" or "COQ6" or "COQ7" or "COQ8A" or "COQ8B" or "COQ9" or "COX10" or "COX14" or "COX15" or "COX16" or "COX20" or "COX4I1" or "COX4I2" or "COX5A" or "COX6A1" or "COX6A2" or "COX6B1" or "COX7B" or "COX8A" or "CP" or "CPOX" or "CPS1" or "CPT1A" or "CPT1C" or "CPT2" or "CRAT" or "CRPPA" or "CSGALNACT1" or "CTH" or "CTNS" or "CTPS1" or "CTSA" or "CTSB" or "CTSC" or "CTSD" or "CTSF" or "CTSK" or	1164890

"CUBN" or "CYB561" or "CYB5A" or "CYB5R3" or "CYC1" or "CYCS" or "CYP11A1" or "CYP11B1" or "CYP11B2" or "CYP17A1" or "CYP19A1" or "CYP21A2" or "CYP27A1" or "CYP2U1" or "CYP4F22" or "CYP51A1" or "CYP7A1" or "CYP7B1" or "D2HGDH" or "DALRD3" or "DARS1" or "DARS2" or "DBH" or "DBT" or "DCXR" or "DDC" or "DDHD1" or "DDHD2" or "DDOST" or "DEGS1" or "DGAT1" or "DGKE" or "DGUOK" or "DHCR24" or "DHCR7" or "DHDDS" or "DHFR" or "DHODH" or "DHTKD1" or "DIABLO" or "DKC1" or "DLAT" or "DLD" or "DLST" or "DMGDH" or "DNA2" or "DNAJC12" or "DNAJC19" or "DNAJC21" or "DNAJC5" or "DNAJC6" or "DNM1" or "DNM1L" or "DNM2" or "DOLK " or "DPAGT1" or "DPM1" or "DPM2" or "DPM3" or "DPYD" or "DPYS" or "DSE" or "DTNBP1" or "DTYMK" or "DUT" or "DYM" or "DYNC1H1" or "EARS2" or "EBP" or "ECHS1" or "EFL1" or "EHHADH" or "EIF6" or "ELAC2" or "ELOVL1" or "ELOVL4" or "ELOVL4" or "ELOVL5" or "ELP1" or "ELP2" or "EMC1" or "EMG1" or "ENO3" or "ENPP1" or "ENTPD1" or "EOGT" or "EPG5" or "EPHX1" or "EPM2A" or "EPRS1" or "ERAL1" or "ESR1" or "ESR2" or "ETFA" or "ETFB" or "ETFDH" or "ETHE1" or "EXT1" or "EXT2" or "EXT2" or "EXTL3" or "FA2H" or "FAAH2" or "FAH" or "FAM20B" or "FAR1" or "FARS2" or "FARSA" or "FARSB" or "FASTKD2" or "FBP1" or "FBXL4" or "FCSK" or "FDFT1" or "FDPS" or "FDX2" or "FDXR" or "FECH" or "FH" or "FIG4" or "FKRP" or "FKTN" or "FLAD1" or "FMO3" or "FOLR1" or "FOXRED1" or "FTCD" or "FTH1" or "FTL" or "FTSJ1" or "FUCA1" or "FUT8" or "FXN" or "G6PC" or "G6PC3" or "G6PD" or "GAA" or "GABBR2" or "GABRA1" or "GABRA6" or "GABRB1" or "GABRB2" or "GABRB3" or "GABRD" or "GABRG2" or "GAD1" or "GALC" or "GALE" or "GALK1" or "GALM" or "GALNS" or "GALNT14" or "GALNT3" or "GALT" or "GAMT" or "GANAB" or "GARS1" or "GATA1" or "GATB" or "GATC" or "GATM" or "GATM" or "GBA" or "GBA2" or "GBE1" or "GCDH" or "GCH1" or "GCK" or "GCLC" or "GDAP1" or "GFER" or "GFM1" or "GFM2" or "GFPT1" or "GFUS" or "GGCX" or "GGPS1" or "GGT1" or "GK" or "GLA" or "GLB1" or "GLB1" or "GLDC" or "GLRA1" or "GLRB" or "GLRX5" or "GLS" or "GLUD1" or "GLUL" or "GLYCTK" or "GM2A" or "GMPPA" or "GMPPB" or "GNE" or "GNE" or "GNMT" or "GNPAT" or "GNPNAT1" or "GNPTAB" or "GNPTG" or "GNS" or "GON7" or "GORAB" or "GOSR2" or "GOT2" or "GPAA1" or "GPD1" or "GPHN" or "GPI" or "GPIHBP1" or "GPT2" or "GPX4" or "GRHPR" or "GRIA2" or "GRIA3" or "GRIA4" or "GRID2" or "GRIN1" or "GRIN2A" or "GRIN2B" or "GRIN2D" or "GRM1" or "GRM6" or "GRN" or "GSR" or "GSS" or "GSTZ1" or "GTPBP3" or "GUF1" or "GUSB" or "GYG1" or "GYG2" or "GYS1" or "GYS2" or "H6PD" or "HAAO" or "HACD1" or "HADH" or "HADHA" or "HADHB" or "HAL" or "HAMP" or "HAO1" or "HARS1" or "HARS2" or "HCCS" or "HCFC1" or "HEPHL1" or "HEXA" or "HEXB" or "HFE" or "HGD" or "HGSNAT" or "HIBADH" or "HIBCH" or "HJV" or "HK1" or "HLCS" or "HMBS" or "HMGCL" or "HMGCS2" or "HMOX1" or "HNF1A" or "HNF1B" or "HNF4A" or "HOGA1" or "HPD" or "HPGD" or "HPRT1" or "HPS1" or "HPS3" or "HPS4" or "HPS5" or "HPS6" or "HS6ST1" or "HS6ST2" or "HSD11B1" or "HSD11B2" or "HSD17B10" or "HSD17B3" or "HSD17B4" or "HSD3B2" or "HSD3B7" or "HSPA9" or "HSPD1" or "HSPE1" or "HTRA2" or "HYAL1" or "HYKK" or "IARS1" or "IARS2" or "IBA57" or "IDH1" or "IDH2" or "IDH3A" or "IDH3B" or "IDS" or "IDUA" or "IFIH1" or "IL1RAPL1" or "IMPDH1" or "INPP5E" or "INPP5K" or "INPPL1" or "INS" or "INSR" or "ISCA1" or "ISCA2" or "ISCU" or "ITPA" or "ITPR1" or "ITPR2" or "IVD" or "JAGN1" or "KARS1" or "KCNJ11" or "KCTD7" or "KDSR" or "KHK" or "KIF1A" or "KIF5A" or "KIF5C" or "KLF11" or "KMO" or "KYNU" or "L2HGDH" or "LACC1" or "LAGE3" or "LAMP2" or "LAP3" or "LARGE1" or "LARS1" or "LARS2" or "LBR" or "LCAT" or "LCT" or "LDHA" or "LDHB" or "LDHD" or "LDLR" or "LDLRAP1" or "LFNG" or "LIAS" or "LIPA" or "LIPC" or "LIPE" or "LIPH" or "LIPN" or "LIPT1" or "LIPT2" or "LMAN1" or "LMBRD1" or "LMF1" or "LONP1" or "LPA" or "LPAR6" or "LPIN1" or "LPIN2" or "LPL" or "LRPPRC" or "LRRK2" or "LSS" or "LTC4S" or "LYRM4" or "LYRM7" or "LYST" or "MAGT1" or

"MAN1B1" or "MAN2B1" or "MAN2B2" or "MANBA" or "MAOA" or "MAOB" or "MARS1" or "MARS2" or "MAT1A" or "MAT2A" or "MBOAT7" or "MBTPS1" or "MC2R" or "MCAT" or "MCCC1" or "MCCC2" or "MCEE" or "MCFD2" or "MCOLN1" or "MDH1" or "MDH2" or "MECR" or "MFF" or "MFN2" or "MFSD2A" or "MFSD8" or "MGAT2" or "MGME1" or "MICOS13" or "MICU1" or "MICU2" or "MIEF2" or "MIPEP" or "MLPH" or "MLYCD" or "MMAA" or "MMAB" or "MMACHC" or "MMADHC" or "MMUT" or "MOCOS" or "MOCS1" or "MOCS2" or "MOCS3" or "MOGS" or "MPC1" or "MPDU1" or "MPI" or "MPST" or "MPV17" or "MRAP" or "MRM2" or "MRPL12" or "MRPL24" or "MRPL3" or "MRPL44" or "MRPS14" or "MRPS16" or "MRPS2" or "MRPS22" or "MRPS23" or "MRPS25" or "MRPS28" or "MRPS34" or "MRPS7" or "MSMO1" or "MSTO1" or "MTAP" or "MT-ATP6" or "MT-ATP8" or "MT-CO1" or "MT-CO2" or "MT-CO3" or "MT-CYB" or "MTFMT" or "MTHFD1" or "MTHFR" or "MTHFS" or "MTM1" or "MTMR2" or "MT-ND1" or "MT-ND2" or "MT-ND3" or "MT-ND4" or "MT-ND4L" or "MT-ND5" or "MT-ND6" or "MTO1" or "MTPAP" or "MTR" or "MT-RNR1" or "MT-RNR2" or "MTRR" or "MT-TA" or "MT-TC" or "MT-TD" or "MT-TE" or "MT-TF" or "MT-TG" or "MT-TH" or "MT-TI" or "MT-TK" or "MT-TL1" or "MT-TL2" or "MT-TM" or "MT-TN" or "MTTP" or "MT-TP" or "MT-TQ" or "MT-TR" or "MT-TS1" or "MT-TS2" or "MT-TT" or "MT-TV" or "MT-TW" or "MT-TY" or "MVD" or "MVK" or "MYO5A" or "NADK2" or "NADSYN1" or "NAGA" or "NAGLU" or "NAGS" or "NANS" or "NAPB" or "NARS1" or "NARS2" or "NAT8L" or "NAXD" or "NAXE" or "NBAS" or "NBEAL2" or "NDST1" or "NDUFA1" or "NDUFA10" or "NDUFA11" or "NDUFA12" or "NDUFA13" or "NDUFA2" or "NDUFA4" or "NDUFA6" or "NDUFA8" or "NDUFA9" or "NDUFAF1" or "NDUFAF2" or "NDUFAF3" or "NDUFAF4" or "NDUFAF5" or "NDUFAF6" or "NDUFAF7" or "NDUFAF8" or "NDUFB10" or "NDUFB11" or "NDUFB3" or "NDUFB7" or "NDUFB8" or "NDUFB9" or "NDUFC2" or "NDUFS1" or "NDUFS2" or "NDUFS3" or "NDUFS4" or "NDUFS6" or "NDUFS7" or "NDUFS8" or "NDUFV1" or "NDUFV2" or "NEPRO" or "NEU1" or "NEUROD1" or "NFE2L2" or "NFS1" or "NFU1" or "NGLY1" or "NHLRC1" or "NME3" or "NMNAT1" or "NNT" or "NOLA2" or "NOLA3" or "NPC1" or "NPC2" or "NPL" or "NPM1" or "NR1H4" or "NR3C1" or "NR3C2" or "NSDHL" or "NSUN2" or "NSUN3" or "NT5C3A" or "NT5E" or "NUBPL" or "NUDT15" or "NUS1" or "OAS1" or "OAT" or "OCRL" or "ODC1" or "OGDH" or "OGT" or "OPA1" or "OPA3" or "OPLAH" or "OSGEP" or "OSTC" or "OTC" or "OXA1L" or "OXCT1" or "PAH" or "PAICS" or "PAM16" or "PANK2" or "PAPSS2" or "PARN" or "PARS2" or "PAX4" or "PC" or "PCBD1" or "PCCA" or "PCCB" or "PCK1" or "PCK2" or "PCSK1" or "PCSK9" or "PCSK9" or "PCYT1A" or "PCYT1A" or "PCYT2" or "PDE12" or "PDHA1" or "PDHB" or "PDHX" or "PDK3" or "PDP1" or "PDPR" or "PDSS1" or "PDSS2" or "PDX1" or "PDXK" or "PDZK1IP1" or "PEPD" or "PET100" or "PET117" or "PEX1" or "PEX10" or "PEX11B" or "PEX12" or "PEX13" or "PEX14" or "PEX16" or "PEX19" or "PEX2" or "PEX26" or "PEX3" or "PEX5" or "PEX5" or "PEX6" or "PEX7" or "PFKM" or "PGAM2" or "PGAP1" or "PGAP2" or "PGAP3" or "PGK1" or "PGM1" or "PGM3" or "PGR" or "PHGDH" or "PHKA1" or "PHKA2" or "PHKB" or "PHKG2" or "PHYH" or "PHYKPL" or "PI4K2A" or "PI4KA" or "PIGA" or "PIGB" or "PIGC" or "PIGG" or "PIGH" or "PIGK" or "PIGL" or "PIGM" or "PIGN" or "PIGO" or "PIGP" or "PIGQ" or "PIGS" or "PIGT" or "PIGU" or "PIGV" or "PIGW" or "PIGY" or "PIK3C2A" or "PIK3CA" or "PIK3CD" or "PIK3R1" or "PIK3R2" or "PIK3R5" or "PIKFYVE" or "PINK1" or "PIP5K1C" or "PISD" or "PITRM1" or "PKLR" or "PLA2G4A" or "PLA2G6" or "PLCB1" or "PLCB3" or "PLCB4" or "PLCD1" or "PLCE1" or "PLCG2" or "PLIN1" or "PLIN5" or "PLPBP" or "PMM2" or "PMPCA" or "PMPCB" or "PMVK" or "PNKD" or "PNP" or "PNPLA1" or "PNPLA2" or "PNPLA4" or "PNPLA6" or "PNPLA8" or "PNPO" or "PNPT1" or "POFUT1" or "POGLUT1" or "POLG" or "POLG2" or "POLR1A" or "POLR1B" or "POLR1C" or "POLR1D" or "POLR3A" or "POLR3B" or "POLR3H" or "POLRMT" or "POMGNT1" or

"POMGNT2" or "POMK" or "POMT1" or "POMT2" or "POP1" or "POR" or "PPA2" or "PPCS" or "PPM1K" or "PPOX" or "PPT1" or "PRDX1" or "PRKAG2" or "PRKCSH" or "PRKN" or "PRODH" or "PRODH2" or "PRORP" or "PRPS1" or "PRRT2" or "PSAP" or "PSAT1" or "PSPH" or "PSTPIP1" or "PTCD3" or "PTDSS1" or "PTEN" or "PTRH2" or "PTS" or "PUS1" or "PUS3" or "PYCR1" or "PYCR2" or "PYGL" or "PYGM" or "QARS1" or "QDPR" or "QRSL1" or "RAB18" or "RAB23" or "RAB27A" or "RAB3GAP1" or "RAB3GAP2" or "RAB7A" or "RARS1" or "RARS2" or "RBCK1" or "RBSN" or "RFT1" or "RFX6" or "RIC3" or "RMND1" or "RMRP" or "RNASEH1" or "RNASEH2A" or "RNASEH2B" or "RNASEH2C" or "RNASET2" or "RNF31" or "RPIA" or "RPL10" or "RPL11" or "RPL13" or "RPL15" or "RPL18" or "RPL21" or "RPL26" or "RPL27" or "RPL35" or "RPL35A" or "RPL5" or "RPS10" or "RPS15A" or "RPS17" or "RPS19" or "RPS20" or "RPS23" or "RPS24" or "RPS26" or "RPS27" or "RPS28" or "RPS29" or "RPS7" or "RPSA" or "RRM2B" or "RTN4IP1" or "RUBCN" or "RXYLT1" or "SACS" or "SAMHD1" or "SAR1B" or "SARDH" or "SARS1" or "SARS2" or "SAT1" or "SBDS" or "SBF1" or "SBF2" or "SC5D" or "SCARB1" or "SCARB2" or "SCO1" or "SCO2" or "SCP2" or "SCYL1" or "SCYL2" or "SDHA" or "SDHA" or "SDHAF1" or "SDHAF2" or "SDHB" or "SDHB" or "SDHC" or "SDHD" or "SDR9C7" or "SEC23A" or "SEC23B" or "SECISBP2" or "SELENBP1" or "SELENOI" or "SEPSECS" or "SERAC1" or "SFXN4" or "SGMS2" or "SGPL1" or "SGSH" or "SHMT2" or "SHPK" or "SI" or "SLC10A1" or "SLC10A2" or "SLC10A7" or "SLC11A2" or "SLC13A3" or "SLC13A5" or "SLC16A1" or "SLC16A1" or "SLC17A5" or "SLC18A2" or "SLC19A1" or "SLC19A2" or "SLC19A3" or "SLC1A1" or "SLC1A2" or "SLC1A3" or "SLC1A4" or "SLC22A12" or "SLC22A5" or "SLC25A1" or "SLC25A10" or "SLC25A11" or "SLC25A12" or "SLC25A13" or "SLC25A15" or "SLC25A19" or "SLC25A20" or "SLC25A21" or "SLC25A22" or "SLC25A24" or "SLC25A26" or "SLC25A3" or "SLC25A32" or "SLC25A38" or "SLC25A4" or "SLC25A42" or "SLC25A46" or "SLC26A1" or "SLC26A2" or "SLC27A4" or "SLC27A5" or "SLC28A1" or "SLC29A1" or "SLC29A3" or "SLC2A1" or "SLC2A10" or "SLC2A2" or "SLC2A9" or "SLC30A10" or "SLC30A2" or "SLC30A9" or "SLC33A1" or "SLC35A1" or "SLC35A2" or "SLC35A3" or "SLC35C1" or "SLC35D1" or "SLC36A2" or "SLC36A2 " or "SLC37A4" or "SLC38A8" or "SLC39A13" or "SLC39A14" or "SLC39A4" or "SLC39A8" or "SLC3A1" or "SLC40A1" or "SLC45A1" or "SLC46A1" or "SLC52A1" or "SLC52A2" or "SLC52A3" or "SLC5A1" or "SLC5A2" or "SLC5A6" or "SLC5A7" or "SLC6A1" or "SLC6A17" or "SLC6A19" or "SLC6A2" or "SLC6A20" or "SLC6A3" or "SLC6A5" or "SLC6A6" or "SLC6A8" or "SLC6A9" or "SLC7A14" or "SLC7A2" or "SLC7A3" or "SLC7A5" or "SLC7A7" or "SLC7A9" or "SLC9A7" or "SLCO1B1" or "SLCO1B3" or "SLCO2A1" or "SMPD1" or "SMPD4" or "SMS" or "SNAP25" or "SNAP29" or "SNORD118" or "SNX14" or "SORCS3" or "SORD" or "SPATA5" or "SPG11" or "SPG20" or "SPG7" or "SPNS2" or "SPR" or "SPTLC1" or "SPTLC2" or "SQOR" or "SRD5A2" or "SRD5A3" or "SSBP1" or "SSR3" or "SSR4" or "ST3GAL3" or "ST3GAL5" or "STAP1" or "STAR" or "STAT2" or "STEAP3" or "STS" or "STT3A" or "STT3B" or "STX11" or "STX1B" or "STXBP1" or "STXBP2" or "SUCLA2" or "SUCLG1" or "SUGCT" or "SULT2B1" or "SUMF1" or "SUOX" or "SURF1" or "SV2A" or "SYN1" or "SYNJ1" or "SYT1" or "SYT14" or "SYT2" or "TACO1" or "TAF1A" or "TALDO1" or "TANGO2" or "TARS1" or "TARS2" or "TAT" or "TAZ" or "TBC1D24" or "TBK1" or "TBXAS1" or "TCN1" or "TCN2" or "TCOF1" or "TDO2" or "TECPR2" or "TECR" or "TF" or "TFAM" or "TFR2" or "TFRC" or "TH" or "THAP11" or "THG1L" or "TIMM22" or "TIMM50" or "TIMM8A" or "TIMMDC1" or "TK2" or "TKFC" or "TKT" or "TLCD3B" or "TMEM126A" or "TMEM126B" or "TMEM165" or "TMEM173" or "TMEM199" or "TMEM70" or "TMLHE" or "TMPRSS6" or "TOMM70" or "TOP3A" or "TOR1A" or "TP53RK" or "TPI1" or "TPK1" or "TPMT" or "TPP1" or "TPRKB" or "TRAK1" or "TRAPPC11" or "TRAPPC12" or "TRAPPC2" or "TRAPPC2L" or "TRAPPC4" or "TRAPPC6B" or "TRAPPC9" or "TREH" or "TREX1" or "TRIP11" or "TRIT1" or "TRMT1"

	or "TRMT10A" or "TRMT10C" or "TRMT5" or "TRMU" or "TRNT1" or "TSEN15" or "TSEN2" or "TSEN34" or "TSEN54" or "TSFM" or "TSR2" or "TTC19" or "TTPA" or "TUFM" or "TUSC3" or "TWNK" or "TXN2" or "TXNRD2" or "TYMP" or "TYR" or "UBIAD1" or "UBTF" or "UCP2" or "UGCG" or "UGDH" or "UGP2" or "UGT1A1" or "UMPS" or "UNC13D" or "UNG" or "UPB1" or "UQCC2" or "UQCC3" or "UQCRB" or "UQCRC2" or "UQCRFS1" or "UQCRQ" or "UROC1" or "UROD" or "UROS" or "VAC14" or "VAMP1" or "VAMP2" or "VAPB" or "VARS1" or "VARS2" or "VIPAS39" or "VKORC1" or "VLDLR" or "VMA21" or "VPS11" or "VPS13A" or "VPS13B" or "VPS13C" or "VPS13D" or "VPS33A" or "VPS33B" or "VPS45" or "VPS4A" or "WARS1" or "WARS2" or "WDR4" or "WDR45" or "XDH" or "XPNPEP3" or "XYLT1" or "XYLT2" or "YARS1" or "YARS2" or "YIF1B" or "YME1L1" or "YRDC" or "ZFYVE26" or "ZNF143").ab,kf. [genes]	
7	((metabolic adj2 result?) or blood or heamtol* or urin* or uret* or liquor or sweat or Perspiration or enzym? or fibroblasts or leucocyte? or protein? or biopsy or fibroblasts).mp.	9682513
8	phenotyp*.mp.	744808
9	exp neurodevelopmental disorders/	197773
10	(neurodevelopmental adj3 (abnormalit* or error? or syndrome? or disorder? or disease? or deficiency)).mp.	18180
11	Mitochondrial Diseases/ge [Genetics]	2942
12	((gene? or genom* or genetic*) and mitochondria?).mp.	170120
13	exp mitochondrial diseases/ and ge.fs.	9872
14	((metabolic or metabolism or neurodevelopmental) adj3 (abnormalit* or error?)).mp.	42074
15	or/7-14 [unexplained phenotypes/metabolic errors]	10239734
16	and/4-6	3775
17	and/4-5,15	13430
18	and/4,6,15	7168
19	or/16-18	18141
20	exp animals/ not humans/	4959724
21	19 not 20	17092

22	(Strain or Culture or "resistance mechanism" or Clone? or antibiotic or "Peseudomonas aruginosa" or virulence or "Sequence type" or coli or bacteri* or amr or colistin or carbapenemase or (host not (host adj3 cell)) or colonization or fluroquinolone or "ceftazide vibactam" or "resistant isolate" or "beta lactamase" or MCT or gentamicin or esbt or klebsielle pneumonia or tetracycline or "antimicrobial resistance gene" or pig or MRSA or "Resistance mechanism" or aureus or serotype or outbreak or "bloodstream infection" or chicken or "human infection" or organism).mp. [VOS green cluster 2]	3803998
23	(chemotherap* or tumo?r? or tp53 or melanoma* or tumorigen* or metastas* or (cancer? not (cancer? adj5 (inborn or inheret* or heredit* or familial))) or sarcoma or brca1 or pten or brca2).mp. or neoplasm?.ab,ti. [VOS blue cluster 3]	3799780
24	(plant or (tree not (family tree or pedigree tree or decision tree)) or soil or genus or "phylogenetic analysis" or fruit or "complete chloroplast genome").mp. [VOS yellow cluster 4]	1172752
25	("Mycobacterium tuberculosis" or rifampicine or (tuberculosis not (tuberculosis adj3 (treatment or therapy or intervention))) or "resistant mutant" or "cross resistance").mp. [VOS purple cluster 5]	253631
26	or/22-25 [VOS cluster 1 no exclusions]	8210372
27	21 not 26	10634

Ovid Embase Classic+Embase <1947 to 2022 February 15>
Search date: 16 February 2022

#	Searches	Results
1	exp whole genome sequencing/ or whole exome sequencing/	61421
2	(wgs or (whole genom* adj5 sequenc*)).ab,kw,ti.	37479
3	(wes or ((exome or Transcriptome) adj3 sequenc*)).ab,kw,ti.	51471
4	(genome adj3 sequenc*).mp.	103778
5	(erratum or correction).ti. or erratum.pt.	298040
6	4 and 5	536
7	or/1-3,6 [WES/WGS]	105884
8	di.fs. or (predict: or specificity).tw. or diagnosis.ab.	7454473

9	("A4GALT" or "AAGAB" or "AARS1" or "AARS2" or "AASS" or "ABAT" or "ABCA1" or "ABCA12" or "ABCB11" or "ABCB4" or "ABCB6" or "ABCB7" or "ABCC2" or "ABCC6" or "ABCC8" or "ABCC8" or "ABCD1" or "ABCD3" or "ABCD4" or "ABCG5" or "ABCG8" or "ABHD12" or "ABHD5" or "ACACA" or "ACACB" or "ACAD8" or "ACAD9" or "ACADM" or "ACADS" or "ACADSB" or "ACADVL" or "ACAT1" or "ACAT2" or "ACBD5" or "ACER3" or "ACO2" or "ACOX1" or "ACOX2" or "ACSF3" or "ACSL4" or "ACY1" or "ADA" or "ADA2" or "ADAR" or "ADARB1" or "ADAT3" or "ADCK2" or "ADK" or "ADSL" or "ADSS1" or "AFG3L2" or "AGA" or "AGK" or "AGL" or "AGMO" or "AGPAT2" or "AGPS" or "AGXT" or "AGXT2" or "AHCY" or "AICDA" or "AIFM1" or "AIMP1" or "AIMP2" or "AK1" or "AK2" or "AK7" or "AKR1C2" or "AKR1D1" or "AKT2" or "ALAD" or "ALAS2" or "ALAS2" or "ALB" or "ALDH18A1" or "ALDH1L2" or "ALDH3A2" or "ALDH4A1" or "ALDH5A1" or "ALDH6A1" or "ALDH7A1" or "ALDOA" or "ALDOB" or "ALG1" or "ALG11" or "ALG12" or "ALG13" or "ALG14" or "ALG2" or "ALG3" or "ALG6" or "ALG8" or "ALG9" or "ALOX12B" or "ALOXE3" or "ALPL" or "AMACR" or "AMN" or "AMPD1" or "AMPD2" or "AMPD3" or "AMT" or "ANGPTL3" or "ANPEP" or "AP1B1" or "AP1S1" or "AP1S2" or "AP2S1" or "AP3B1" or "AP3B2" or "AP3D1" or "AP4B1" or "AP4E1" or "AP4M1" or "AP4S1" or "AP5Z1" or "APOA1" or "APOA5" or "APOB" or "APOC2" or "APOC3" or "APOE" or "APOE" or "APOO" or "APOPT1" or "APPL1" or "APRT" or "AR" or "ARCN1" or "ARFGEF2" or "ARG1" or "ARSA" or "ARSB" or "ARSG" or "ASAH1" or "ASL" or "ASNS" or "ASPA" or "ASS1" or "ATAD1" or "ATAD3A" or "ATG5" or "ATIC" or "ATP13A2" or "ATP5F1A" or "ATP5F1D" or "ATP5F1E" or "ATP5MD" or "ATP5PO" or "ATP6AP1" or "ATP6AP2" or "ATP6V0A2" or "ATP6V1A" or "ATP6V1E1" or "ATP7A" or "ATP7B" or "ATP8A2" or "ATP8B1" or "ATPAF2" or "AUH" or "B3GALNT2" or "B3GALT6" or "B3GAT3" or "B3GLCT" or "B4GALNT1" or "B4GALT1" or "B4GALT7" or "B4GAT1" or "BAAT" or "BBOX1" or "BCAP31" or "BCAT2" or "BCKDHA" or "BCKDHB" or "BCKDK" or "BCS1L" or "BLK" or "BLOC1S3" or "BLOC1S6" or "BLVRA" or "BMP6" or "BMS1" or "BOLA3" or "BPNT2" or "BSCL2" or "BSCL2" or "BTD" or "C12orf65" or "C1GALT1C1" or "C1QBP" or "CA5A" or "CAD" or "CANT1" or "CARS1" or "CARS2" or "CAT" or "CBLIF" or "CBS" or "CCDC115" or "CCS" or "CD320" or "CEP89" or "CERS1" or "CERS2" or "CERS3" or "CETP" or "CHAT" or "CHCHD10" or "CHCHD2" or "CHKB" or "CHRNE" or "CHST11" or "CHST14" or "CHST3" or "CHST6" or "CHSY1" or "CLCN2" or "CLN3" or "CLN5" or "CLN6" or "CLN8" or "CLP1" or "CLPB" or "CLPP" or "CLPX" or "CLTC" or "CMPK2" or "CNDP1" or "COA3" or "COA5" or "COA6" or "COA7" or "COASY" or "COG1" or "COG2" or "COG4" or "COG5" or "COG6" or "COG7" or "COG8" or "COL4A3BP" or "COPA" or "COPB2" or "COQ2" or "COQ4" or "COQ5" or "COQ6" or "COQ7" or "COQ8A" or "COQ8B" or "COQ9" or "COX10" or "COX14" or "COX15" or "COX16" or "COX20" or "COX4I1" or "COX4I2" or "COX5A" or "COX6A1" or "COX6A2" or "COX6B1" or "COX7B" or "COX8A" or "CP" or "CPOX" or "CPS1" or "CPT1A" or "CPT1C" or "CPT2" or "CRAT" or "CRPPA" or "CSGALNACT1" or "CTH" or "CTNS" or "CTPS1" or "CTSA" or "CTSB" or "CTSC" or "CTSD" or "CTSF" or "CTSK" or "CUBN" or "CYB561" or "CYB5A" or "CYB5R3" or "CYC1" or "CYCS" or "CYP11A1" or "CYP11B1" or "CYP11B2" or "CYP17A1" or "CYP19A1" or "CYP21A2" or "CYP27A1" or "CYP2U1" or "CYP4F22" or "CYP51A1" or "CYP7A1" or "CYP7B1" or "D2HGDH" or "DALRD3" or "DARS1" or "DARS2" or "DBH" or "DBT" or "DCXR" or "DDC" or "DDHD1" or "DDHD2" or "DDOST" or "DEGS1" or "DGAT1" or "DGKE" or "DGUOK" or "DHCR24" or "DHCR7" or "DHDDS" or "DHFR" or "DHODH" or "DHTKD1" or "DIABLO" or "DKC1" or "DLAT" or "DLD" or "DLST" or "DMGDH" or "DNA2" or "DNAJC12" or "DNAJC19" or "DNAJC21" or "DNAJC5" or "DNAJC6" or "DNM1" or "DNM1L" or "DNM2" or "DOLK " or "DPAGT1" or "DPM1" or "DPM2" or "DPM3" or "DPYD" or "DPYS" or "DSE" or "DTNBP1" or "DTYMK" or "DUT" or "DYM" or "DYNC1H1" or "EARS2" or "EBP" or "ECHS1" or "EFL1" or "EHHADH" or "EIF6" or	1713824

"ELAC2" or "ELOVL1" or "ELOVL4" or "ELOVL4" or "ELOVL5" or "ELP1" or "ELP2" or "EMC1" or "EMG1" or "ENO3" or "ENPP1" or "ENTPD1" or "EOGT" or "EPG5" or "EPHX1" or "EPM2A" or "EPRS1" or "ERAL1" or "ESR1" or "ESR2" or "ETFA" or "ETFB" or "ETFDH" or "ETHE1" or "EXT1" or "EXT2" or "EXT2" or "EXTL3" or "FA2H" or "FAAH2" or "FAH" or "FAM20B" or "FAR1" or "FARS2" or "FARSA" or "FARSB" or "FASTKD2" or "FBP1" or "FBXL4" or "FCSK" or "FDFT1" or "FDPS" or "FDX2" or "FDXR" or "FECH" or "FH" or "FIG4" or "FKRP" or "FKTN" or "FLAD1" or "FMO3" or "FOLR1" or "FOXRED1" or "FTCD" or "FTH1" or "FTL" or "FTSJ1" or "FUCA1" or "FUT8" or "FXN" or "G6PC" or "G6PC3" or "G6PD" or "GAA" or "GABBR2" or "GABRA1" or "GABRA6" or "GABRB1" or "GABRB2" or "GABRB3" or "GABRD" or "GABRG2" or "GAD1" or "GALC" or "GALE" or "GALK1" or "GALM" or "GALNS" or "GALNT14" or "GALNT3" or "GALT" or "GAMT" or "GANAB" or "GARS1" or "GATA1" or "GATB" or "GATC" or "GATM" or "GATM" or "GBA" or "GBA2" or "GBE1" or "GCDH" or "GCH1" or "GCK" or "GCLC" or "GDAP1" or "GFER" or "GFM1" or "GFM2" or "GFPT1" or "GFUS" or "GGCX" or "GGPS1" or "GGT1" or "GK" or "GLA" or "GLB1" or "GLB1" or "GLDC" or "GLRA1" or "GLRB" or "GLRX5" or "GLS" or "GLUD1" or "GLUL" or "GLYCTK" or "GM2A" or "GMPPA" or "GMPPB" or "GNE" or "GNE" or "GNMT" or "GNPAT" or "GNPNAT1" or "GNPTAB" or "GNPTG" or "GNS" or "GON7" or "GORAB" or "GOSR2" or "GOT2" or "GPAA1" or "GPD1" or "GPHN" or "GPI" or "GPIHBP1" or "GPT2" or "GPX4" or "GRHPR" or "GRIA2" or "GRIA3" or "GRIA4" or "GRID2" or "GRIN1" or "GRIN2A" or "GRIN2B" or "GRIN2D" or "GRM1" or "GRM6" or "GRN" or "GSR" or "GSS" or "GSTZ1" or "GTPBP3" or "GUF1" or "GUSB" or "GYG1" or "GYG2" or "GYS1" or "GYS2" or "H6PD" or "HAAO" or "HACD1" or "HADH" or "HADHA" or "HADHB" or "HAL" or "HAMP" or "HAO1" or "HARS1" or "HARS2" or "HCCS" or "HCFC1" or "HEPHL1" or "HEXA" or "HEXB" or "HFE" or "HGD" or "HGSNAT" or "HIBADH" or "HIBCH" or "HJV" or "HK1" or "HLCS" or "HMBS" or "HMGCL" or "HMGCS2" or "HMOX1" or "HNF1A" or "HNF1B" or "HNF4A" or "HOGA1" or "HPD" or "HPGD" or "HPRT1" or "HPS1" or "HPS3" or "HPS4" or "HPS5" or "HPS6" or "HS6ST1" or "HS6ST2" or "HSD11B1" or "HSD11B2" or "HSD17B10" or "HSD17B3" or "HSD17B4" or "HSD3B2" or "HSD3B7" or "HSPA9" or "HSPD1" or "HSPE1" or "HTRA2" or "HYAL1" or "HYKK" or "IARS1" or "IARS2" or "IBA57" or "IDH1" or "IDH2" or "IDH3A" or "IDH3B" or "IDS" or "IDUA" or "IFIH1" or "IL1RAPL1" or "IMPDH1" or "INPP5E" or "INPP5K" or "INPPL1" or "INS" or "INSR" or "ISCA1" or "ISCA2" or "ISCU" or "ITPA" or "ITPR1" or "ITPR2" or "IVD" or "JAGN1" or "KARS1" or "KCNJ11" or "KCTD7" or "KDSR" or "KHK" or "KIF1A" or "KIF5A" or "KIF5C" or "KLF11" or "KMO" or "KYNU" or "L2HGDH" or "LACC1" or "LAGE3" or "LAMP2" or "LAP3" or "LARGE1" or "LARS1" or "LARS2" or "LBR" or "LCAT" or "LCT" or "LDHA" or "LDHB" or "LDHD" or "LDLR" or "LDLRAP1" or "LFNG" or "LIAS" or "LIPA" or "LIPC" or "LIPE" or "LIPH" or "LIPN" or "LIPT1" or "LIPT2" or "LMAN1" or "LMBRD1" or "LMF1" or "LONP1" or "LPA" or "LPAR6" or "LPIN1" or "LPIN2" or "LPL" or "LRPPRC" or "LRRK2" or "LSS" or "LTC4S" or "LYRM4" or "LYRM7" or "LYST" or "MAGT1" or "MAN1B1" or "MAN2B1" or "MAN2B2" or "MANBA" or "MAOA" or "MAOB" or "MARS1" or "MARS2" or "MAT1A" or "MAT2A" or "MBOAT7" or "MBTPS1" or "MC2R" or "MCAT" or "MCCC1" or "MCCC2" or "MCEE" or "MCFD2" or "MCOLN1" or "MDH1" or "MDH2" or "MECR" or "MFF" or "MFN2" or "MFSD2A" or "MFSD8" or "MGAT2" or "MGME1" or "MICOS13" or "MICU1" or "MICU2" or "MIEF2" or "MIPEP" or "MLPH" or "MLYCD" or "MMAA" or "MMAB" or "MMACHC" or "MMADHC" or "MMUT" or "MOCOS" or "MOCS1" or "MOCS2" or "MOCS3" or "MOGS" or "MPC1" or "MPDU1" or "MPI" or "MPST" or "MPV17" or "MRAP" or "MRM2" or "MRPL12" or "MRPL24" or "MRPL3" or "MRPL44" or "MRPS14" or "MRPS16" or "MRPS2" or "MRPS22" or "MRPS23" or "MRPS25" or "MRPS28" or "MRPS34" or "MRPS7" or "MSMO1" or "MSTO1" or "MTAP" or "MT-ATP6" or "MT-

ATP8" or "MT-CO1" or "MT-CO2" or "MT-CO3" or "MT-CYB" or "MTFMT" or "MTHFD1" or "MTHFR" or "MTHFS" or "MTM1" or "MTMR2" or "MT-ND1" or "MT-ND2" or "MT-ND3" or "MT-ND4" or "MT-ND4L" or "MT-ND5" or "MT-ND6" or "MTO1" or "MTPAP" or "MTR" or "MT-RNR1" or "MT-RNR2" or "MTRR" or "MT-TA" or "MT-TC" or "MT-TD" or "MT-TE" or "MT-TF" or "MT-TG" or "MT-TH" or "MT-TI" or "MT-TK" or "MT-TL1" or "MT-TL2" or "MT-TM" or "MT-TN" or "MTTP" or "MT-TP" or "MT-TQ" or "MT-TR" or "MT-TS1" or "MT-TS2" or "MT-TT" or "MT-TV" or "MT-TW" or "MT-TY" or "MVD" or "MVK" or "MYO5A" or "NADK2" or "NADSYN1" or "NAGA" or "NAGLU" or "NAGS" or "NANS" or "NAPB" or "NARS1" or "NARS2" or "NAT8L" or "NAXD" or "NAXE" or "NBAS" or "NBEAL2" or "NDST1" or "NDUFA1" or "NDUFA10" or "NDUFA11" or "NDUFA12" or "NDUFA13" or "NDUFA2" or "NDUFA4" or "NDUFA6" or "NDUFA8" or "NDUFA9" or "NDUFAF1" or "NDUFAF2" or "NDUFAF3" or "NDUFAF4" or "NDUFAF5" or "NDUFAF6" or "NDUFAF7" or "NDUFAF8" or "NDUFB10" or "NDUFB11" or "NDUFB3" or "NDUFB7" or "NDUFB8" or "NDUFB9" or "NDUFC2" or "NDUFS1" or "NDUFS2" or "NDUFS3" or "NDUFS4" or "NDUFS6" or "NDUFS7" or "NDUFS8" or "NDUFV1" or "NDUFV2" or "NEPRO" or "NEU1" or "NEUROD1" or "NFE2L2" or "NFS1" or "NFU1" or "NGLY1" or "NHLRC1" or "NME3" or "NMNAT1" or "NNT" or "NOLA2" or "NOLA3" or "NPC1" or "NPC2" or "NPL" or "NPM1" or "NR1H4" or "NR3C1" or "NR3C2" or "NSDHL" or "NSUN2" or "NSUN3" or "NT5C3A" or "NT5E" or "NUBPL" or "NUDT15" or "NUS1" or "OAS1" or "OAT" or "OCRL" or "ODC1" or "OGDH" or "OGT" or "OPA1" or "OPA3" or "OPLAH" or "OSGEP" or "OSTC" or "OTC" or "OXA1L" or "OXCT1" or "PAH" or "PAICS" or "PAM16" or "PANK2" or "PAPSS2" or "PARN" or "PARS2" or "PAX4" or "PC" or "PCBD1" or "PCCA" or "PCCB" or "PCK1" or "PCK2" or "PCSK1" or "PCSK9" or "PCSK9" or "PCYT1A" or "PCYT1A" or "PCYT2" or "PDE12" or "PDHA1" or "PDHB" or "PDHX" or "PDK3" or "PDP1" or "PDPR" or "PDSS1" or "PDSS2" or "PDX1" or "PDXK" or "PDZK1IP1" or "PEPD" or "PET100" or "PET117" or "PEX1" or "PEX10" or "PEX11B" or "PEX12" or "PEX13" or "PEX14" or "PEX16" or "PEX19" or "PEX2" or "PEX26" or "PEX3" or "PEX5" or "PEX5" or "PEX6" or "PEX7" or "PFKM" or "PGAM2" or "PGAP1" or "PGAP2" or "PGAP3" or "PGK1" or "PGM1" or "PGM3" or "PGR" or "PHGDH" or "PHKA1" or "PHKA2" or "PHKB" or "PHKG2" or "PHYH" or "PHYKPL" or "PI4K2A" or "PI4KA" or "PIGA" or "PIGB" or "PIGC" or "PIGG" or "PIGH" or "PIGK" or "PIGL" or "PIGM" or "PIGN" or "PIGO" or "PIGP" or "PIGQ" or "PIGS" or "PIGT" or "PIGU" or "PIGV" or "PIGW" or "PIGY" or "PIK3C2A" or "PIK3CA" or "PIK3CD" or "PIK3R1" or "PIK3R2" or "PIK3R5" or "PIKFYVE" or "PINK1" or "PIP5K1C" or "PISD" or "PITRM1" or "PKLR" or "PLA2G4A" or "PLA2G6" or "PLCB1" or "PLCB3" or "PLCB4" or "PLCD1" or "PLCE1" or "PLCG2" or "PLIN1" or "PLIN5" or "PLPBP" or "PMM2" or "PMPCA" or "PMPCB" or "PMVK" or "PNKD" or "PNP" or "PNPLA1" or "PNPLA2" or "PNPLA4" or "PNPLA6" or "PNPLA8" or "PNPO" or "PNPT1" or "POFUT1" or "POGLUT1" or "POLG" or "POLG2" or "POLR1A" or "POLR1B" or "POLR1C" or "POLR1D" or "POLR3A" or "POLR3B" or "POLR3H" or "POLRMT" or "POMGNT1" or "POMGNT2" or "POMK" or "POMT1" or "POMT2" or "POP1" or "POR" or "PPA2" or "PPCS" or "PPM1K" or "PPOX" or "PPT1" or "PRDX1" or "PRKAG2" or "PRKCSH" or "PRKN" or "PRODH" or "PRODH2" or "PRORP" or "PRPS1" or "PRRT2" or "PSAP" or "PSAT1" or "PSPH" or "PSTPIP1" or "PTCD3" or "PTDSS1" or "PTEN" or "PTRH2" or "PTS" or "PUS1" or "PUS3" or "PYCR1" or "PYCR2" or "PYGL" or "PYGM" or "QARS1" or "QDPR" or "QRSL1" or "RAB18" or "RAB23" or "RAB27A" or "RAB3GAP1" or "RAB3GAP2" or "RAB7A" or "RARS1" or "RARS2" or "RBCK1" or "RBSN" or "RFT1" or "RFX6" or "RIC3" or "RMND1" or "RMRP" or "RNASEH1" or "RNASEH2A" or "RNASEH2B" or "RNASEH2C" or "RNASET2" or "RNF31" or "RPIA" or "RPL10" or "RPL11" or "RPL13" or "RPL15" or "RPL18" or "RPL21" or "RPL26" or "RPL27" or "RPL35" or "RPL35A" or "RPL5" or "RPS10" or "RPS15A" or "RPS17" or "RPS19" or

"RPS20" or "RPS23" or "RPS24" or "RPS26" or "RPS27" or "RPS28" or "RPS29" or "RPS7" or "RPSA" or "RRM2B" or "RTN4IP1" or "RUBCN" or "RXYLT1" or "SACS" or "SAMHD1" or "SAR1B" or "SARDH" or "SARS1" or "SARS2" or "SAT1" or "SBDS" or "SBF1" or "SBF2" or "SC5D" or "SCARB1" or "SCARB2" or "SCO1" or "SCO2" or "SCP2" or "SCYL1" or "SCYL2" or "SDHA" or "SDHA" or "SDHAF1" or "SDHAF2" or "SDHB" or "SDHB" or "SDHC" or "SDHD" or "SDR9C7" or "SEC23A" or "SEC23B" or "SECISBP2" or "SELENBP1" or "SELENOI" or "SEPSECS" or "SERAC1" or "SFXN4" or "SGMS2" or "SGPL1" or "SGSH" or "SHMT2" or "SHPK" or "SI" or "SLC10A1" or "SLC10A2" or "SLC10A7" or "SLC11A2" or "SLC13A3" or "SLC13A5" or "SLC16A1" or "SLC16A1" or "SLC17A5" or "SLC18A2" or "SLC19A1" or "SLC19A2" or "SLC19A3" or "SLC1A1" or "SLC1A2" or "SLC1A3" or "SLC1A4" or "SLC22A12" or "SLC22A5" or "SLC25A1" or "SLC25A10" or "SLC25A11" or "SLC25A12" or "SLC25A13" or "SLC25A15" or "SLC25A19" or "SLC25A20" or "SLC25A21" or "SLC25A22" or "SLC25A24" or "SLC25A26" or "SLC25A3" or "SLC25A32" or "SLC25A38" or "SLC25A4" or "SLC25A42" or "SLC25A46" or "SLC26A1" or "SLC26A2" or "SLC27A4" or "SLC27A5" or "SLC28A1" or "SLC29A1" or "SLC29A3" or "SLC2A1" or "SLC2A10" or "SLC2A2" or "SLC2A9" or "SLC30A10" or "SLC30A2" or "SLC30A9" or "SLC33A1" or "SLC35A1" or "SLC35A2" or "SLC35A3" or "SLC35C1" or "SLC35D1" or "SLC36A2" or "SLC36A2 " or "SLC37A4" or "SLC38A8" or "SLC39A13" or "SLC39A14" or "SLC39A4" or "SLC39A8" or "SLC3A1" or "SLC40A1" or "SLC45A1" or "SLC46A1" or "SLC52A1" or "SLC52A2" or "SLC52A3" or "SLC5A1" or "SLC5A2" or "SLC5A6" or "SLC5A7" or "SLC6A1" or "SLC6A17" or "SLC6A19" or "SLC6A2" or "SLC6A20" or "SLC6A3" or "SLC6A5" or "SLC6A6" or "SLC6A8" or "SLC6A9" or "SLC7A14" or "SLC7A2" or "SLC7A3" or "SLC7A5" or "SLC7A7" or "SLC7A9" or "SLC9A7" or "SLCO1B1" or "SLCO1B3" or "SLCO2A1" or "SMPD1" or "SMPD4" or "SMS" or "SNAP25" or "SNAP29" or "SNORD118" or "SNX14" or "SORCS3" or "SORD" or "SPATA5" or "SPG11" or "SPG20" or "SPG7" or "SPNS2" or "SPR" or "SPTLC1" or "SPTLC2" or "SQOR" or "SRD5A2" or "SRD5A3" or "SSBP1" or "SSR3" or "SSR4" or "ST3GAL3" or "ST3GAL5" or "STAP1" or "STAR" or "STAT2" or "STEAP3" or "STS" or "STT3A" or "STT3B" or "STX11" or "STX1B" or "STXBP1" or "STXBP2" or "SUCLA2" or "SUCLG1" or "SUGCT" or "SULT2B1" or "SUMF1" or "SUOX" or "SURF1" or "SV2A" or "SYN1" or "SYNJ1" or "SYT1" or "SYT14" or "SYT2" or "TACO1" or "TAF1A" or "TALDO1" or "TANGO2" or "TARS1" or "TARS2" or "TAT" or "TAZ" or "TBC1D24" or "TBK1" or "TBXAS1" or "TCN1" or "TCN2" or "TCOF1" or "TDO2" or "TECPR2" or "TECR" or "TF" or "TFAM" or "TFR2" or "TFRC" or "TH" or "THAP11" or "THG1L" or "TIMM22" or "TIMM50" or "TIMM8A" or "TIMMDC1" or "TK2" or "TKFC" or "TKT" or "TLCD3B" or "TMEM126A" or "TMEM126B" or "TMEM165" or "TMEM173" or "TMEM199" or "TMEM70" or "TMLHE" or "TMPRSS6" or "TOMM70" or "TOP3A" or "TOR1A" or "TP53RK" or "TPI1" or "TPK1" or "TPMT" or "TPP1" or "TPRKB" or "TRAK1" or "TRAPPC11" or "TRAPPC12" or "TRAPPC2" or "TRAPPC2L" or "TRAPPC4" or "TRAPPC6B" or "TRAPPC9" or "TREH" or "TREX1" or "TRIP11" or "TRIT1" or "TRMT1" or "TRMT10A" or "TRMT10C" or "TRMT5" or "TRMU" or "TRNT1" or "TSEN15" or "TSEN2" or "TSEN34" or "TSEN54" or "TSFM" or "TSR2" or "TTC19" or "TTPA" or "TUFM" or "TUSC3" or "TWNK" or "TXN2" or "TXNRD2" or "TYMP" or "TYR" or "UBIAD1" or "UBTF" or "UCP2" or "UGCG" or "UGDH" or "UGP2" or "UGT1A1" or "UMPS" or "UNC13D" or "UNG" or "UPB1" or "UQCC2" or "UQCC3" or "UQCRB" or "UQCRC2" or "UQCRFS1" or "UQCRQ" or "UROC1" or "UROD" or "UROS" or "VAC14" or "VAMP1" or "VAMP2" or "VAPB" or "VARS1" or "VARS2" or "VIPAS39" or "VKORC1" or "VLDLR" or "VMA21" or "VPS11" or "VPS13A" or "VPS13B" or "VPS13C" or "VPS13D" or "VPS33A" or "VPS33B" or "VPS45" or "VPS4A" or "WARS1" or "WARS2" or "WDR4" or "WDR45" or "XDH" or "XPNPEP3" or "XYLT1" or "XYLT2"

or "YARS1" or "YARS2" or "YIF1B" or "YME1L1" or "YRDC" or "ZFYVE26" or "ZNF143").ab,kw. [genes]

10	((metabolic adj2 result?) or blood or heamtol* or urin* or uret* or liquor or sweat or Perspiration or enzym? or fibroblasts or leucocyte? or protein? or biopsy or fibroblasts).mp.	13261084
11	phenotyp*.mp.	1001015
12	inborn errors of metabolism.kw.	1020
13	(neurodevelopmental adj3 (abnormalit* or error? or syndrome? or disorder? or disease? or deficiency)).mp.	21089
14	((gene? or genom* or genetic*) and mitochondria?).ab,hw,kw,sh,ti.	181262
15	exp "disorders of mitochondrial functions"/ and (phenotype or genetics).mp.	9174
16	((metabolic or metabolism or neurodevelopmental) adj3 (abnormalit* or error?)).mp.	41999
17	or/10-16 [unexplained phenotypes/metabolic errors]	13736394
18	and/7-9	7854
19	and/7-8,17	26715
20	and/7,9,17	15161
21	or/18-20	36396
22	(animal/ or animal experiment/ or animal model/ or nonhuman/ or rat/ or mouse/ or (rat or rats or mouse or mice).ti.) not human/	7315597
23	21 not 22	32254

24	(Strain or Culture or "resistance mechanism" or Clone? or antibiotic or "Peseudomonas aruginosa" or virulence or "Sequence type" or coli or bacteri* or amr or colistin or carbapenemase or (host not (host adj3 cell)) or colonization or fluroquinolone or "ceftazide vibactam" or "resistant isolate" or "beta lactamase" or MCT or gentamicin or esbt or klebsielle pneumonia or tetracycline or "antimicrobial resistance gene" or pig or MRSA or "Resistance mechanism" or aureus or serotype or outbreak or "bloodstream infection" or chicken or "human infection" or organism).mp. [VOS green cluster 2]	5522301
25	(chemotherap* or tumo?r? or tp53 or melanoma* or tumorigen* or metastas* or (cancer? not (cancer? adj5 (inborn or inheret* or heredit* or familial))) or sarcoma or brca1 or pten or brca2).mp. or neoplasm?.ab,ti. [VOS blue cluster 3]	6122457
26	(plant or (tree not (family tree or pedigree tree or decision tree)) or soil or genus or "phylogenetic analysis" or fruit or "complete chloroplast genome").mp. [VOS yellow cluster 4]	1485594
27	("Mycobacterium tuberculosis" or rifampicine or (tuberculosis not (tuberculosis adj3 (treatment or therapy or intervention))) or "resistant mutant" or "cross resistance").mp. [VOS purple cluster 5]	328923
28	or/24-27 [VOS cluster 1 no exclusions]	12018551
29	23 not 28	18050
30	limit 29 to exclude medline journals	2428
