# Supplementary figures and images for: Inherited metabolic disorders in adults: systematic review on patient characteristics and diagnostic yield of broad sequencing techniques (exome and genome sequencing)
Source: Front Neurol. 2023 Jul 25;14:1206106. doi: 10.3389/fneur.2023.1206106 (PMC10408679; doi:10.3389/fneur.2023.1206106)

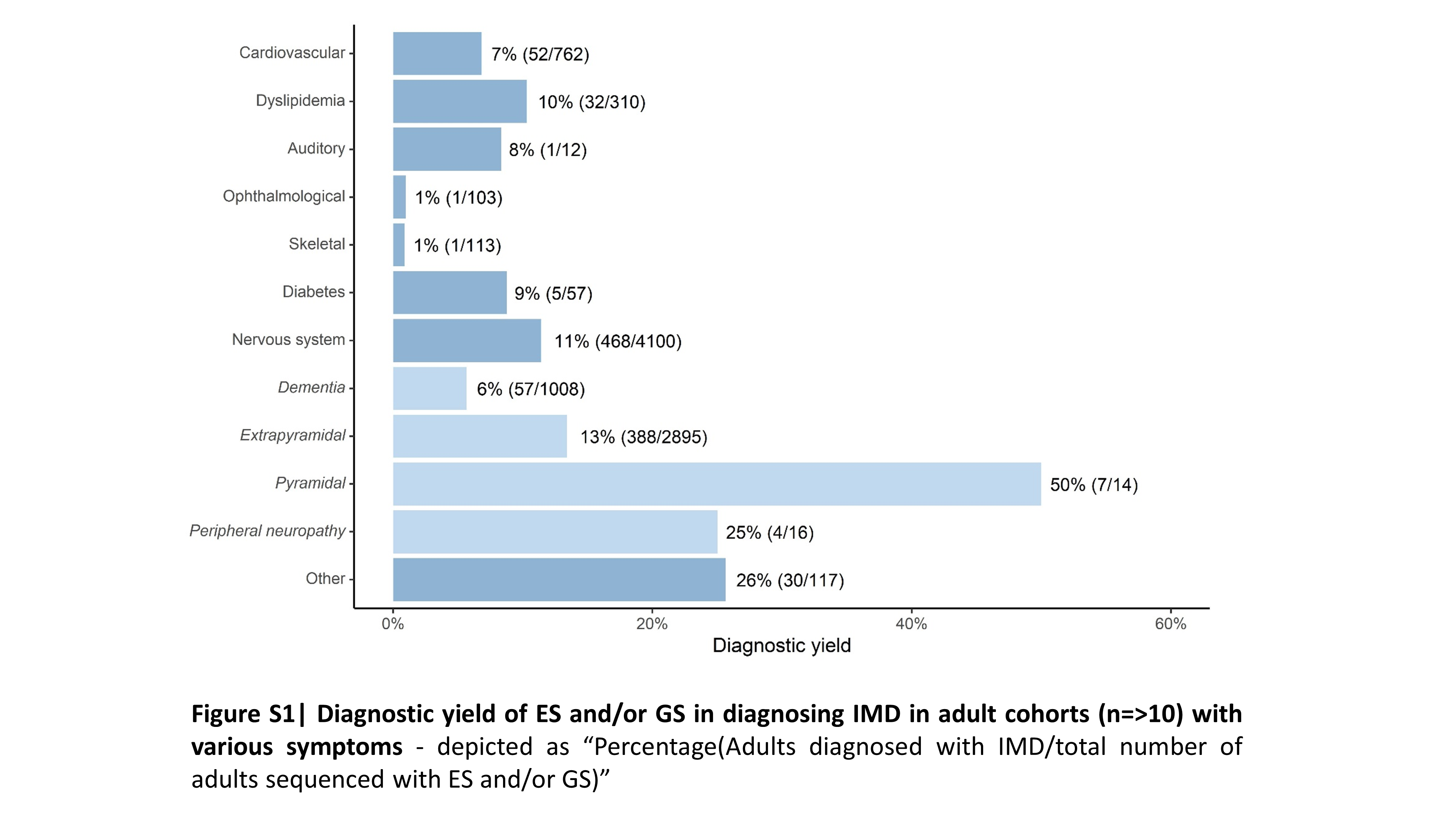

Supplement: Supplementary file 4 [file Image_1.tif]
